# Identification of novel genes that regulate androgen receptor signaling and growth of androgen-deprived prostate cancer cells

**DOI:** 10.18632/oncotarget.3743

**Published:** 2015-04-23

**Authors:** Elina Levina, Hao Ji, Mengqiang Chen, Mirza Baig, David Oliver, Patrice Ohouo, Chang-uk Lim, Garry Schools, Steven Carmack, Ye Ding, Eugenia V. Broude, Igor B. Roninson, Ralph Buttyan, Michael Shtutman

**Affiliations:** ^1^ Department of Drug Discovery and Biomedical Sciences, South Carolina College of Pharmacy, University of South Carolina, Columbia, SC, USA; ^2^ Department of Biological Sciences, University of South Carolina, Columbia, SC, USA; ^3^ Wadsworth Center, NY State Department of Health, Albany, NY, USA; ^4^ The Vancouver Prostate Centre, Vancouver, BC, Canada; ^5^ Cancer Center, Ordway Research Institute, Albany, NY, USA

**Keywords:** prostate cancer, androgen receptor, shRNA, IGSF8, tumor progression

## Abstract

Prostate cancer progression to castration refractory disease is associated with anomalous transcriptional activity of the androgen receptor (AR) in an androgen-depleted milieu. To identify novel gene products whose downregulation transactivates AR in prostate cancer cells, we performed a screen of enzymatically-generated shRNA lenti-libraries selecting for transduced LNCaP cells with elevated expression of a fluorescent reporter gene under the control of an AR-responsive promoter. The shRNAs present in selected populations were analyzed using high-throughput sequencing to identify target genes. Highly enriched gene targets were then validated with siRNAs against selected genes, testing first for increased expression of luciferase from an AR-responsive promoter and then for altered expression of endogenous androgen-regulated genes in LNCaP cells. We identified 20 human genes whose silencing affected the expression of exogenous and endogenous androgen-responsive genes in prostate cancer cells grown in androgen-depleted medium. Knockdown of four of these genes upregulated the expression of endogenous AR targets and siRNAs targeting two of these genes (IGSF8 and RTN1) enabled androgen-independent proliferation of androgen-dependent cells. The effects of IGSF8 appear to be mediated through its interaction with a tetraspanin protein, CD9, previously implicated in prostate cancer progression. Remarkably, homozygous deletions of IGSF8 are found almost exclusively in prostate cancers but not in other cancer types. Our study shows that androgen independence can be achieved through the inhibition of specific genes and reveals a novel set of genes that regulate AR signaling in prostate cancers.

## INTRODUCTION

Metastatic prostate cancer (PCa) patients are treated with androgen deprivation therapies (ADT) to deplete systemic androgen levels and inhibit androgen binding to the androgen receptor (AR) protein in prostate cancer (PCa) cells [1]. ADT extends survival of patients with metastatic disease but most treated patients will eventually recur with castration refractory prostate cancer (CRPC) that continues to grow and metastasize. Although ADT maintains systemic castrate levels of androgen, studies of CRPC tissues and experimental models of CRPC support the notion that CRPC cells remain addicted to AR-driven signaling. This is reinforced by the clinical success of new anti-androgens such as enzalutamide that significantly increase survival of CRPC patients. Mechanistic explanations for AR hyperactivity in CRPC cells include: 1) increased AR expression (with or without concomitant AR gene amplification); 2) AR mutations that allow promiscuous activation by non-androgens; 3) expression of constitutively active truncated AR splice variants produced by alternate splicing [2]; 4) intratumoral steroidogenesis that generates a local tumor microenvironment enriched for testosterone/dihydrotestosterone; 5) tumor cell overexpression of AR co-activators that could further sensitize ARs in CRPC to castrate level androgens or alternate steroids.

Here we considered the hypothesis that AR is inherently capable of functioning under ADT but is functionally suppressed by some other proteins. Reduced expression of such proteins, a new class of PCa-specific tumor suppressors, might facilitate progression to CRPC. To identify genes that may act in this fashion, we developed a shRNA lenti-library screening procedure to identify and characterize genes, the knockdown of which increases the androgen-independent (AI) activity of AR in PCa cells. We combined a reporter-based selection procedure in which shRNAs from transcriptome-scale, enzymatically-generated libraries activate an AR-responsive reporter in androgen-dependent (AD) PCa cells (LNCaP) under the conditions of androgen depletion. Our strategy involved three sequential validation steps following the initial screening. First, we tested siRNAs against gene targets that were enriched following initial selection for the ability to increase the expression of an AR-responsive reporter in androgen-depleted medium. siRNAs that were validated at this step were further tested for effects on the expression of a panel of endogenous AR-regulated genes in androgen-deprived cells. Finally, siRNAs that passed this validation were tested for their ability to enable androgen-independent growth of the cells. Using this protocol, we have identified both known and novel effectors of androgen signaling in androgen-deprived PCa cells. We further demonstrated that some of these genes, when inhibited, permit AI growth of AD cells, demonstrating for the first time that increased AR signaling and AI growth can be achieved through the inhibition of specific genes. Based on published studies, two of the AR signaling modulators identified in our screening, IGSF8 and CD9, appear to interact with each other in PCa progression.

## RESULTS

### shRNA library screening for activation of an AR-responsive promoter

**Figure 1:**
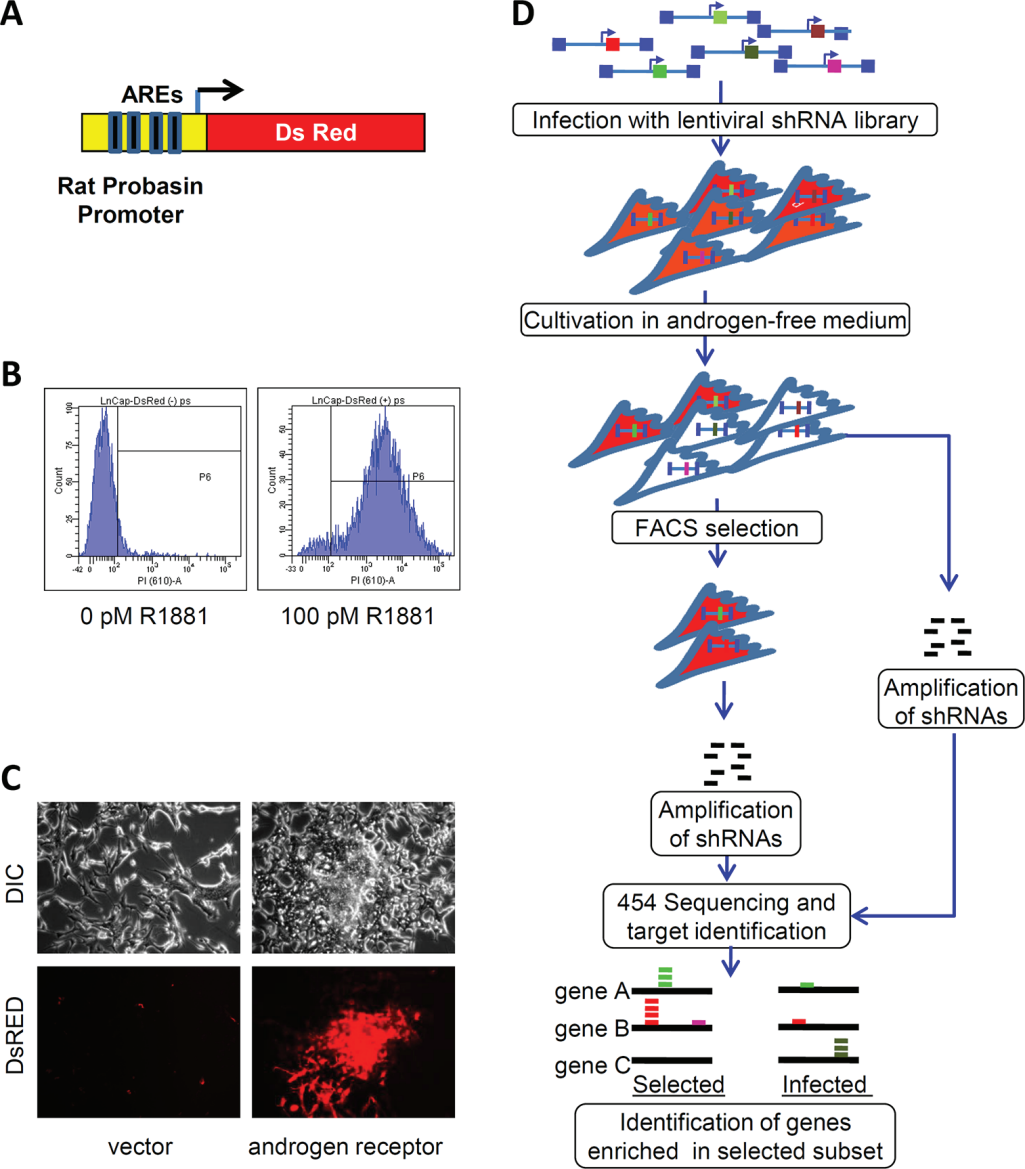
High throughput screening for shRNAs stimulating AR activity in low androgen environment **A.** AR-driven reporter construct containing Red fluorescent protein (DsRed II) under control of modified rat probasin promoter. **B.** FACS analysis of Prb-DsRed-LNCaP reporter cells. Cells were cultured in a hormone-free medium for 8 days (left). Cells were cultured in a hormone-free medium for 6 days followed by 48 h treatment with 100pm R881 (right). **C.** DsRed fluorescence is increased in Prb-DsRed-LNCaP reporter cells infected with AR-expressing (right) or a control lentiviral vector (left) and cultured for 8 days in hormone-free conditions. DIC (top) and fluorescence (bottom) microscopy, 20x magnification. **D.** Scheme of the selection and analysis of the shRNAs stimulating rat probasin promoter under hormone-free conditions.

LNCaP cells were stably transduced with a recombinant lentiviral vector expressing red fluorescent protein (DsRed) under the control of a modified rat probasin gene promoter (pARR2PB) [3] (Figure 1A). These cells were first sorted by FACS to enrich for cells with minimal DsRed fluorescence in androgen-depleted medium. Afterwards, they were again sorted in the presence of R1881 to select for cells with high DsRed fluorescence. The final population contained cells with a maximum dynamic range of induction by an AR ligand (Figure 1B). These reporter cells (Prb-DsRed-LNCaP) expressed constitutively high levels of DsRed when transduced with a lentivirus expressing C-terminal truncated (constitutively active) AR, but not after transduction with a control (insert-free) lentivirus (Figure 1C). Two human shRNA libraries were used for our screen. The first library was prepared from a normalized mixture of cDNA from breast cancer cells (2.8 million total clones) and was previously described [4]. The second shRNA library was derived from a normalized mixture of cDNAs from AD LNCaP cells and AI PC3 cells (1.5 million total clones, see *Methods*).

The selection protocol (Figure 1D) is described in detail in *Methods*. Briefly, Prb-DsRed-LNCaP cells were transduced with lentiviral shRNA libraries. DNA from 10% of transduced cells was extracted immediately after transduction and used as a reference control; the remainder were maintained for 10 days in androgen-depleted medium to minimize basal reporter expression. Then, library-transduced Prb-DsRed-LNCaP cells were FACS-sorted for high DsRed expressers that constituted approximately 3% of the infected cells. Genomic DNA from the high DsRed population was extracted, integrated shRNAs were PCR-amplified and sequenced using 454 massive parallel sequencing. BLAST analysis of the results yielded ~20, 000 sequences per dataset with homology to Unigene clusters. We identified 2913 genes that were 4 fold or more enriched in the high DsRed cell populations (summarized in Supplementary Table S1). The sets of genes selected using breast- and prostate-cell-derived libraries were largely non-overlapping, mainly due to the relatively low sequencing depth of the samples. Using this list of enriched genes we performed a comprehensive automated PubMed search to extract all publications relevant to androgen signaling. This search identified 69 genes that include both co-activators and inhibitors of AR-dependent transcriptional activation (Supplementary Table S2).

### Secondary siRNA screening and the effects of screen-selected siRNAs on the expression of endogenous androgen-regulated genes

**Figure 2:**
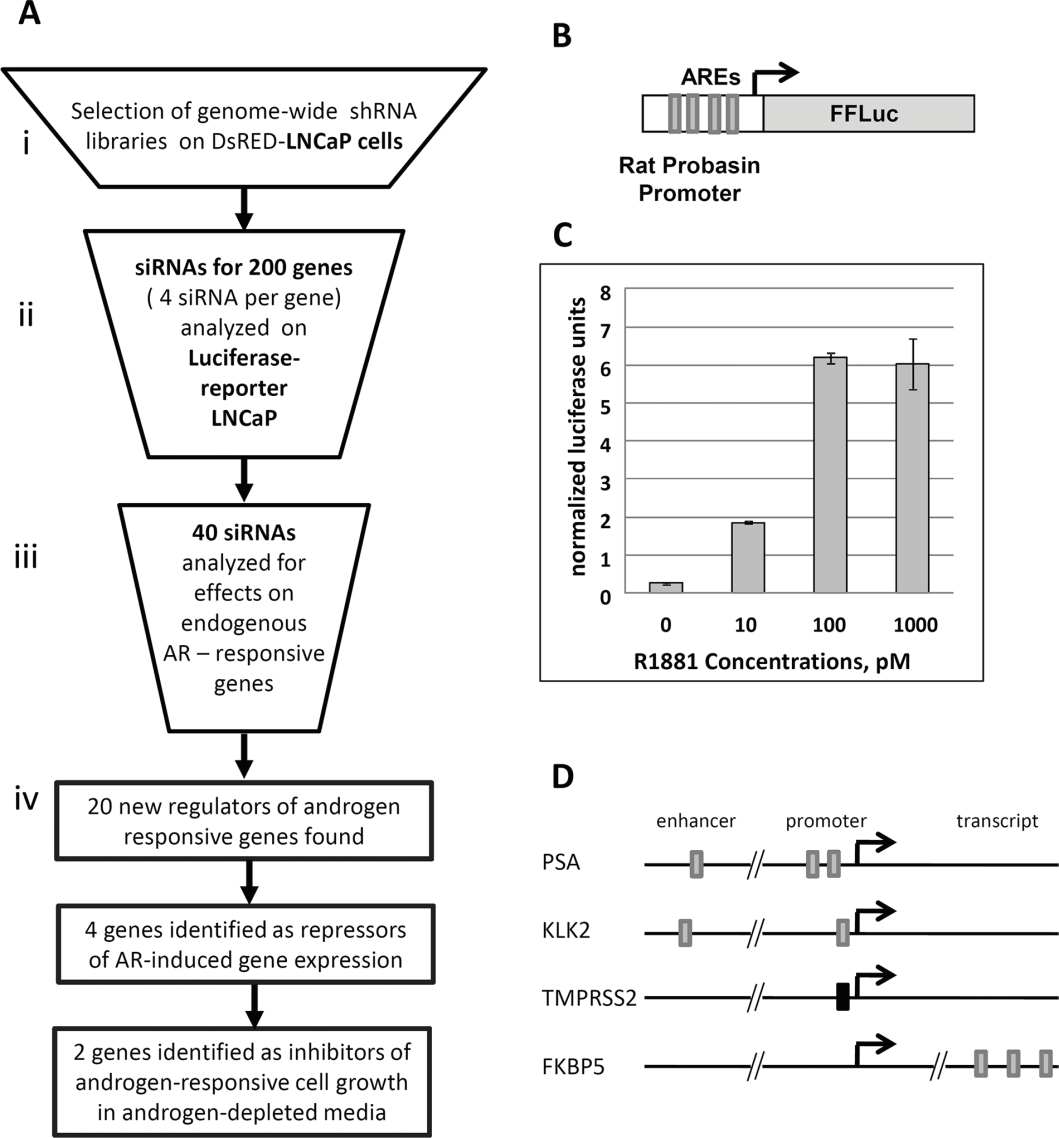
Validation of shRNA library selection procedure **A.** Scheme of library screening and validation procedure. **B.** The AR-driven reporter construct containing Firefly Luciferase (FFLuc) under control of modified rat probasin promoter (validation, step 1) **C.** Androgen dose-dependent activity of the integrated luciferase reporter in Prb-Luc-LNCaP cells **D.** Localization of Androgen Responsive Elements (AREs) in the endogenous genes selected for QPCR based validation. Grey boxes represent canonical AREs, black box represents a non-canonical ARE.

The flow chart of our validation analysis is shown in Figure 2A. Based on the level of enrichment we selected 200 genes (Supplementary Tables S3 and S4) from the primary enriched target list for secondary validation with individual siRNAs (4 different siRNAs per gene). We excluded known androgen signaling regulators and focused on genes that were not previously implicated in androgen signaling. To limit cell line- or reporter-specific effects, we tested siRNAs using a different reporter cell line, LNCaP cells with integrated probasin-luciferase reporter (Prb-Luc-LNCaP, Figure 2B, 2C). Each siRNA was transfected individually into reporter cells that were then maintained in androgen-depleted medium and assayed for luciferase activity. The total amount of DNA in each sample was used to normalize the reporter activity. siRNAs were considered positive in this screen if at least two different siRNAs increased normalized luciferase expression by at least 1.5-fold over control siRNA-transfected cells.

**Figure 3:**
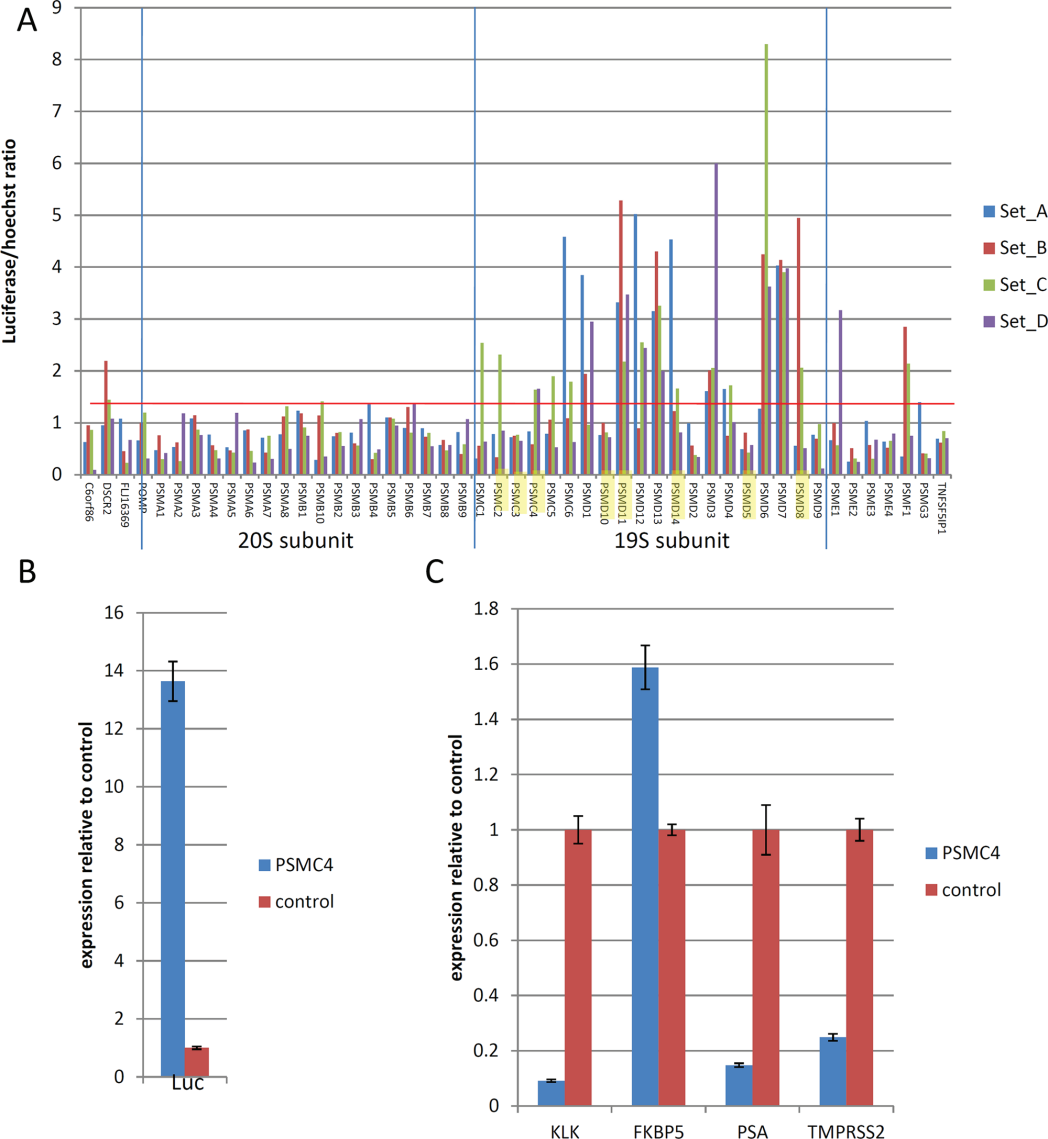
Effects of knockdown of proteasome complex subunits **A.** Effects of siRNAs against the indicated proteasome components (4 siRNA per gene, sets A–D) on Prb-promoter activity measured by luciferase expression in Prb-Luc-LNCaP cells. Genes enriched in shRNA library selection are highlighted. Cells were transfected with siRNAs (5 nM) in duplicates, cultured in hormone-free medium for 6 days, followed by measurement of luciferase activity. **B–C.** Reporter Prb-Luc-LNCaP cells were transfected with siRNA against 19S proteosomal component PSMC4 (5 nM), cultured in hormone-free medium for 6 days, followed by QPCR analysis of expression of firefly luciferase **B.** or endogenous androgen responsive genes **C.** Results represent mean of 3 independent experiments +/– SD.

Among the genes identified by our screen, nine encode different components of the 19S proteasome (highlighted in Figure 3A). It was previously shown that siRNA-mediated disruption of the 19S subunit inhibited AR signaling [5, 6]. Since this was the most enriched class of genes in our selection, we analyzed the effects of the depletion of all the different 20S and 19S proteasome subunits on the activity of the probasin promoter under androgen-free conditions. Whereas all siRNAs targeting 19S subunit genes dramatically increased luciferase reporter expression compared to non-targeting control siRNA, siRNAs targeting 20S subunit genes failed to achieve this effect (Figure 3C). Importantly, depletion of 19S proteins resulted in a 14-fold elevation of luciferase mRNA, confirming the effect on the probasin promoter and arguing against potential stabilization of luciferase protein upon proteasome depletion (Figure 3B). Hence, our selection identified not only individual genes but also specific functionally related classes of genes that regulate the AR-dependent promoter function.

**Figure 4:**
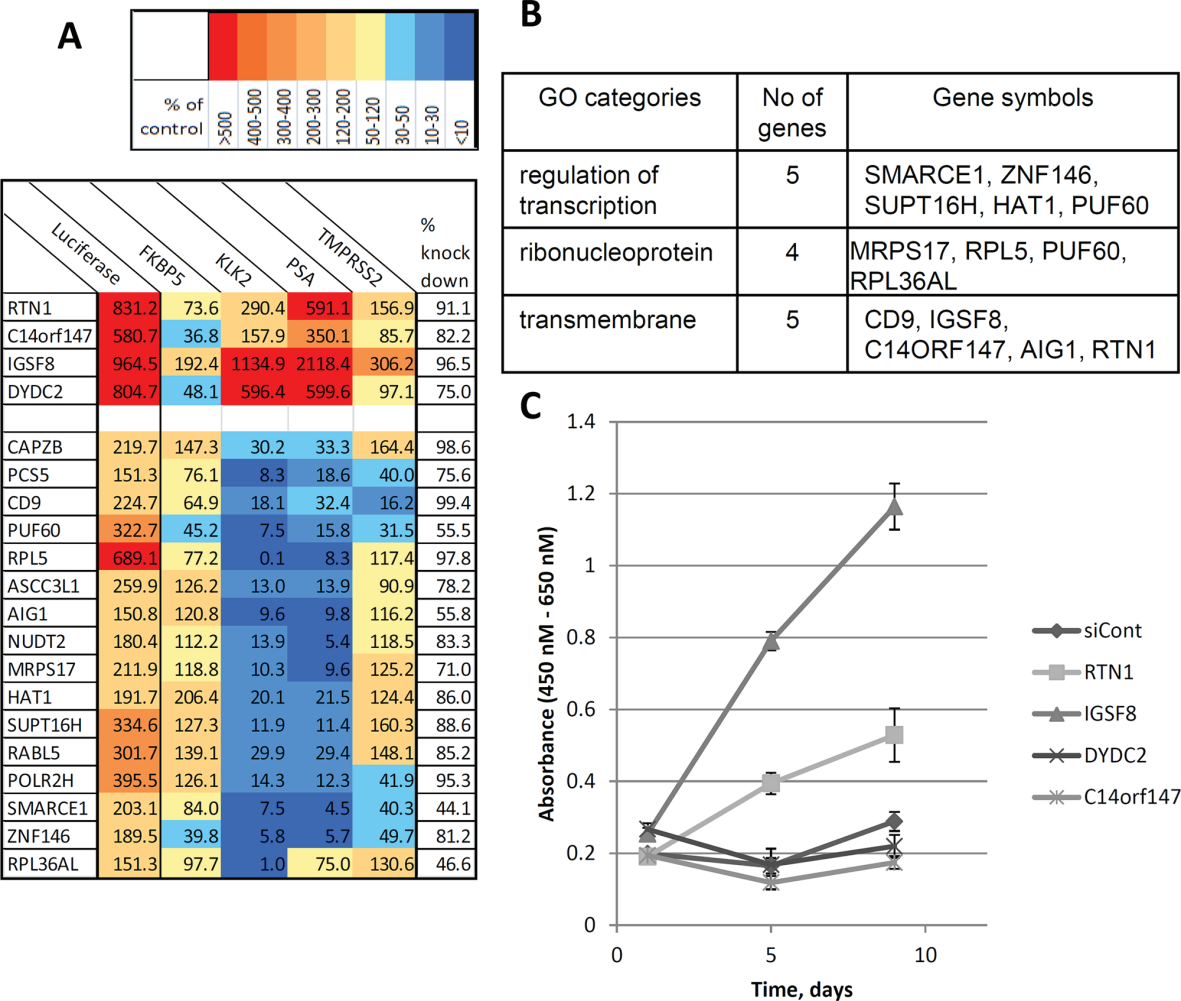
New regulators of androgen-responsive genes identified in the functional screening **A.** Two groups of siRNA regulators were discovered: androgen independent activators and inhibitors of androgen responsive genes. The expression of 4 known androgen-responsive genes (KLF3 (PSA), KLK2, FKBP5 and TMPRSS2) was measured following transfection of selected siRNAs (at 5 nm) into LNCaP cells in triplicates (see Figure 2). Cells were cultured in androgen-free conditions for 6 days. mRNA was purified and subjected to QPCR analysis. Table represents the levels of gene expression as percentages compared to control (the description of genes is presented in Supplementary Table S5). First column represents the effect of selected siRNAs on expression of luciferase in Prb- Luc-LNCaP cells (in triplicate) **B.** Three GO categories enriched in the validated group of genes. **C.** Screen-selected siRNAs enable proliferation of LNCaP cells under hormone-free conditions. LNCaP cells were transfected with siRNAs as shown and cultured under androgen-free conditions. The number of viable cells was determined using spectrophotometric quantification of cell proliferation by WST-1 assay. The plot represents the mean of six independent transfections +/– SD.

40 different siRNA sets with two or more siRNAs that upregulated luciferase expression in reporter cells were selected for the second validation step. Here, we determined the effects of the most active siRNAs for each gene on the expression of 4 endogenous AR-responsive genes with differing regulatory architectures (Figure 2D). We transfected the 40 most active siRNAs as well as a control siRNA into parental (non-reporter) LNCaP cells and, after 6 days androgen-depletion used qPCR to evaluate the expression levels of endogenous AR targets such as KLK2, KLK3 (PSA), TMPRSS2 and FKBP5 (Figure 2A). siRNAs targeting 20 genes significantly affected the expression of endogenous androgen-regulated genes (Figure 4 and Supplementary Table S5) without any significant effect on the expression of AR mRNA (data not shown). Although the magnitude of the effects varied, depending on the specific endogenous gene and siRNA, we grouped the effective siRNAs into two classes: 1) those which mainly induced expression of endogenous androgen-regulated genes (4 siRNAs) and; 2) those which suppressed expression of endogenous genes (16 genes) (Figure 4A). Although siRNAs of both classes induced luciferase expression from the probasin promoter, all four genes in the first class induced the reporter by >5-fold, whereas only one of 16 genes in the second class had such a strong effect on the reporter (Figure 4A). To further confirm the target specificity of the selected siRNAs, we determined the effects of three independent siRNAs targeting PUF60, an RNA binding protein and negative regulator of c-MYC expression, on endogenous AR-regulated genes. All three siRNAs that reduced PUF60 expression (Supplementary Figure S2A) also significantly reduced the expression of KLK2, KLK3 and to a lesser degree, FKBP5, whereas only one siRNA significantly reduced the expression of TMPRSS2 in both androgen-free conditions and in the presence of 100 pM R1881 (Supplementary Figure S2B, S2C).

Since the activity of co-regulators of steroid hormone receptors are often context-dependent [7–9], it is not surprising that the activity of genes that inhibit AR-dependent transactivation of the probasin promoter had different effects on different endogenous genes. Most of the active siRNAs affected mRNA levels of KLK2 and KLK3 (PSA) genes, promoters of which, similar to probasin, contain multiple canonical AREs [5, 11]. In contrast, FKBP5 and TMRSS2 genes, which contain either intronic or non-canonical AREs (Figure 2D) were considerably less affected (Figure 4A) [10, 11]. Therefore, the selection protocol that relies on ARE-driven reporter expression appears to target preferentially endogenous genes with canonical AREs upstream of the transcription initiation site.

### Effects of selected siRNAs on PCa cell growth in androgen deprived medium

We assessed the effects of all four screen-selected siRNAs of the first class (targeting RTN1, IGSF8, C14orf147 (SPTSSA) and DYDC2, Figure 4A) on the growth of LNCaP cells in androgen-deprived medium. Each siRNA was transfected into LNCaP cells that were subsequently maintained in androgen-depleted medium. Two siRNAs, against ISFG8 and RTN1 enabled AD LNCaP cells to grow in androgen-deprived medium while two other siRNAs (C14orf147, DYDC2) and a control non-targeting siRNA had no effect (Figure 4C).

### Effects of IGSF8 knockdown on the expression of androgen receptor-regulated genes

**Figure 5:**
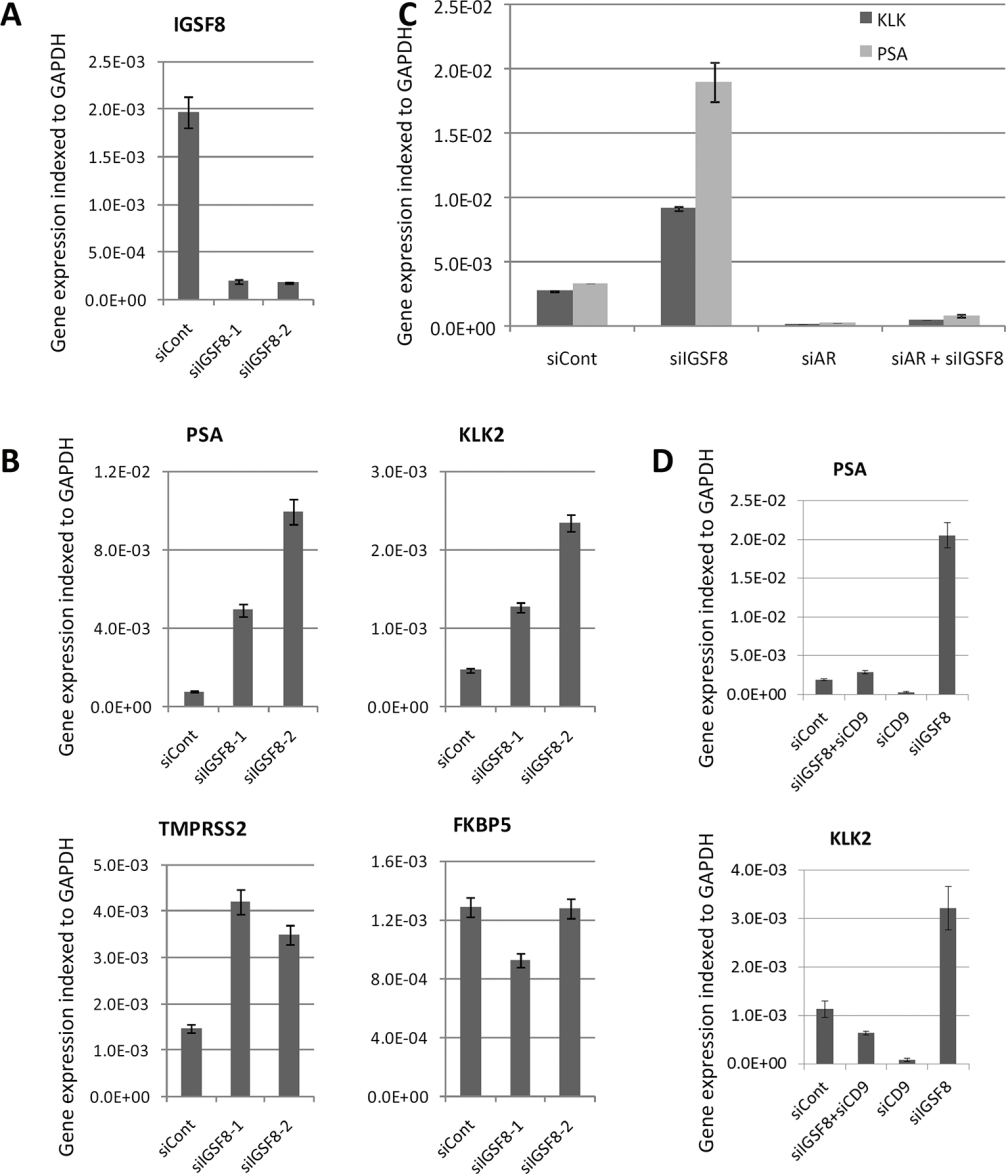
siRNA-mediated depletion of IGSF8 activates the expression of androgen responsive genes **A.** Two different siRNAs efficiently inhibit expression of IGSF8 gene (QPCR analysis). **B.** LNCaP cells were transfected with IGSF8-targeting or control siRNAs (5 nM) and cultured in hormone-free conditions for 6 days. Expression of androgen responsive genes was analyzed by QPCR. The results represents mean of 3 independent experiments +/– SD. **C.** Effect of the IGSF8 knockdown is AR-dependent. LNCaP cells were transfected with control, IGSF8 or AR siRNAs and cultured in hormone-free conditions for 6 days. Expression of androgen responsive genes was analyzed by QPCR. **D.** Effects of the IGSF8 knockdown depend on the expression of CD9. LNCaP cells were transfected with control, IGSF8 or CD9 siRNAs and cultured in hormone-free conditions for 6 days. Expression of androgen responsive genes was analyzed by QPCR. All QPCR results represent mean of 3 independent experiments +/– SD.

**Figure 6:**
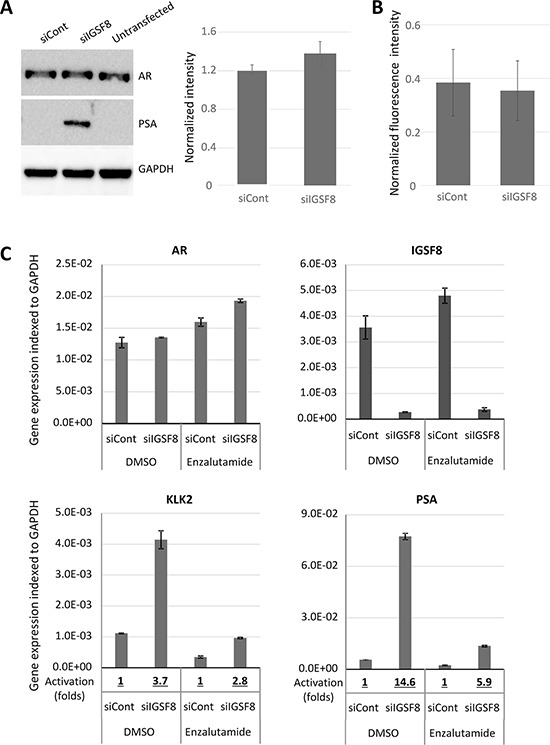
IGSF8 knockdown effects is dependent of AR but does not change AR expression **A.** LNCaP cells were transfected with control or IGSF8-targeting siRNAs (5 nM) and cultured in hormone-free conditions for 6 days. Expression of AR and PSA was analyzed by immunoblotting. Quantification of AR signal intensity normalized to GAPDH signal is shown on the right, relative to AR level of untransfected LNCaP cells (mean of 3 independent experiments +/– SD). **B.** LNCaP were transfected as above, followed by fixation and immunostaining with DAPI and anti-AR antibodies. Images were taken and nuclear staining was quantified. The results represent average quantification of the AR to DAPI ratio of fluorescence intensity (30 nuclei per siRNA) +/– SD. **C.** LNCaP cells were transfected as in A and cultured in hormone-free conditions for 6 days in the presence of 10 μM of Enzalutamide or DMSO. Gene expression was analyzed by QPCR. The results represent mean of 3 independent experiments +/– SD.

Because depletion of IGSF8 conferred the strongest AI growth on LNCaP cells, we analyzed in detail the effect of IGSF8 knockdown on the expression of AR target genes. We designed an independent set of siRNAs against IGSF8, transfected them into LNCaP cells, and determined the mRNA levels of AR target genes after 6 days in androgen-depleted medium. Two different IGSF8-targeting siRNAs yielded greater than 90% IGSF8 mRNA depletion (Figure 5A). Both of these siRNAs also significantly upregulated endogenous mRNA levels of KLK2, KLK3 and TMPRSS2 in androgen-deprived LNCaP cells, while neither had a significant effect on FKBP5 expression (Figure 5B). To test whether AR is involved in IGSF8-dependent activation of endogenous genes we depleted IGSF8 and AR simultaneously. Co-transfection of IGSF8–2 siRNA and AR siRNA suppressed the ability of the IGSF8 siRNA to induce KLK2 and KLK3 expression (Figure 5C) indicating that the effect of IGSF8 was exerted through AR. Several of the siRNAs that affected the expression of AR targets in our screen targeted the tetraspanin protein CD9 that physically interacts with IGSF8 [12]. Curiously, however, CD9 belonged to the second class of AR regulators, as its knockdown decreased the expression of several AR target genes, including KLK2, KLK3 and TMPRSS2 (Figure 4A). As shown in Figure. 5D, siRNA-mediated depletion of CD9 downregulated the expression of KLK2 and KLK3 when used either alone or in combination with IGSF8 shRNA (Figure 5D). To further elucidate the effects of IGSF8 depletion on AR, we analyzed the expression of the AR protein in IGSF8 knockdown cells. IGSF8 knockdown strongly induced PSA expression (Figure 6A), it affected neither the expression (Figure 6A, 6B) nor the nuclear localization of the AR protein (Figure 6B). Finally, the androgen receptor antagonist enzalutamide drastically inhibited the induction of both KLK2 and PSA by IGS8 knockdown, with only minor effects on the expression of AR and IGSF8 (Figure 6C), indicating that the effect of IGSF8 knockdown was mediated by AR.

**Figure 7:**
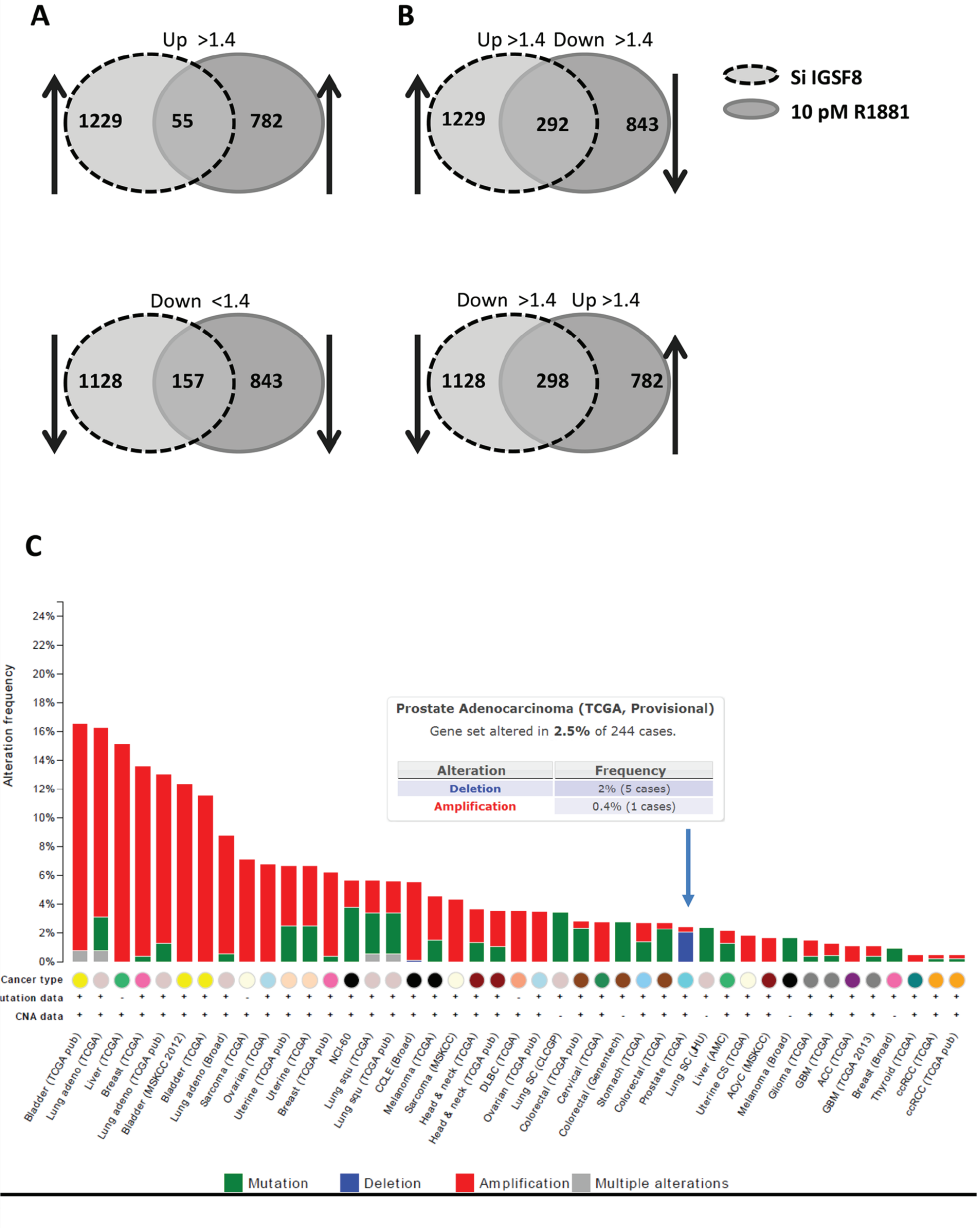
Comparison of gene expression affected by IGSF8 knockdown or androgen stimulation Affymetrix array analysis of gene expression profiles of cells with siRNA knockdown of IGSF8 or treated with R1881. Diagrams represent changes in gene expression > 1.4-fold (*P* < 0.05). **A.** Genes co-regulated by ISF8 knockdown and R1881 treatment. **B.** Genes differentially regulated by IGSF8 siRNA and R1881. **C.** Genetic alterations of IGSF8 in human cancers analyzed through cBioPortal tool (TCGA provisional data set).

Our data show that a subset of known androgen-responsive genes is upregulated by IGSF8 knockdown. To compare the transcriptomic effects of IGSF8 and AR, we analyzed gene expression profiles by microarrays using (i) LNCaP cells with IGSF8 knockdown in androgen-depleted medium; (ii) LNCaP cells stimulated with 10pM of R1881 for 24 hours; (iii) cells transfected with control siRNA in androgen-depleted medium; and (iv) untreated LNCaP cells in androgen-depleted medium. 2357 (7.1%) and 1625 (5%) genes were significantly (>1.4 fold, *P* < 0.05) affected by R1881 treatment or IGSF8 knockdown, respectively. Strikingly, 34% of R1881-regulated genes and 49% of IGSF8 siRNA-responsive genes were regulated by both R1881 and IGSF8 siRNA. 55 genes were upregulated and 157 downregulated by both androgen and IGSF8 knockdown (Figure 7A, Supplementary Table S6). The majority of genes that were induced both by androgen and by IGSF8 shRNA are well-known AR targets, including KLK3(PSA), KLK2, KLK4, PPAP2A, C19orf48, cdc2, and NFKB2 [13–16]. Many AR targets affected by IGSF8 knockdown are known positive and negative regulators of cancer cell proliferation and survival. For example, cdc2 [17–19] and NFKB2 [20–22] enhance androgen-independent growth, and HMGCS2 [23], PIK3AP1 [24], ABCC4 [25], SLC1A5 [26], CYP3A5 [27] genes are associated with PCa progression. Furthermore, many genes downregulated by IGSF8 knockdown are markers of neuroendocrine differentiation (OPRK1 [28, 29], PNMA2 [30], IGFBP3 [31]), cell-adhesion proteins (PCDHB10, PCDHB15, PCDHB8, PCDHB16, PCDHB18, PCDHB12, PCDHB4), targets of AR-regulated transcriptional repressor REST [32, 33], and genes associated with suppression of prostate and other cancers (SERPINI1 [34], ODZ2 [35], SI [36], TLR5 [37, 38], RNF180 [39], FBXL2 [40–42], TRIM45 [43]). A large cohort of genes was differentially regulated by IGSF8 knockdown and androgen (Figure 7B, Supplementary Table S6). These include 292 genes upregulated by IGSF8 knockdown, while downregulated by R881 including pro-oncogenic genes (VAV3 [44–47], REG4 [48, 49], SYP2 [50], ZNF706 [51, 52], SHC4 [53]) and biomarkers of PCa progression (PLA2G2A [54], CLU [55]). 298 genes were downregulated by IGSF8 knockdown while upregulated by R1881 including a cluster of UDP glucuronosyltransferase 2 family genes (UGT2B7, UGT2B17, UGT2B15, UGT2B11, UGT2B10, UGT2B4, UGT2B28, UGT2B7). UGT2B enzymes are mainly responsible for DHT degradation in prostate tissues [56–58]. The main triggers of androgen degradation, UGT2B17 and UGT2B15, were shown to be upregulated by activated AR [59], while they were drastically (>20-fold) downregulated by IGSF8 knockdown. The expression levels of UGT2B17 and UGT2B15 were verified by QPCR in LNCaP with IGSF8 knockdown (with 2 independent siRNAs) (Supplementary Figure S3).

Potential alterations of the IGSF8 gene across various human cancers were analyzed using cBioPortal [60] (Figure 7C). IGSF8 is amplified in a majority of cancer types, except prostate cancers, where homozygous deletions were detected in 2% of analyzed samples (in 5 out of 244 samples of prostate adenocarcinomas) (Figure 7C).

## DISCUSSION

CRPC is thought to be the consequence of dysregulated (hyperactive) androgen signaling in PCa cells that develops subsequent to chronic ADT. In this study, we developed a robust procedure for the identification of new co-regulators of AR that may participate in progression to CRPC. High throughput screens to identify co-regulators of hormone-dependent activation of AR transcriptional activity have been reported [61], but screens for the regulators of AR in hormone-free conditions have, to our knowledge, not been done before. Our procedure employed (i) high-complexity enzymatically generated shRNA libraries that target not only known but also uncharacterized transcripts (both coding and non-coding); (ii) FACS selection of library-infected cells with activated expression of a fluorescent reporter; and (iii) analysis of shRNA enrichment through massive parallel sequencing followed by target identification and validation. The elements of the screening procedure, including target identification through massive parallel sequencing of enzymatically-generated shRNA libraries, were described previously [4]. The combination of the key elements we developed allows for a robust and cost effective screening that can be used for fast identification of co-regulators and the dissection of pathways regulated by any other transcription factors.

Using the multistep screening procedure (Figure 2A), we identified 20 genes, knockdown of which affected the expression of both the probasin-regulated reporter and endogenous androgen-regulated genes. Knockdown of four of these genes results in an upregulation of endogenous AR target genes, while inhibition of the other 16 downregulated endogenous gene expression. The opposite effects of the latter siRNAs on the modified probasin promoter and endogenous genes parallel the reported effects on promoter-dependent activity of previously described co-regulators of AR and other steroid-hormone receptors [9, 62, 63]. In particular, the knockdown of components of the proteosome 19S subunit strongly activates expression from probasin promoter and strongly inhibits the expression of KLK2 and KLK3 in agreement with previous reports [64, 65]. The pleotropic effect of proteosomal inhibition on gene expression causing either upregulation or downregulation of different groups of genes was shown for other steroid hormone receptors (glucocorticoid and estrogen receptors) [66–68].

Our target enrichment list included five genes associated with chromatin remodeling to a transcriptionally active state or with the transcription process (HAT1, SUPT16H and SMARCE1, POLR2H, ZNF146), a cluster of five RNA/ribonucleoprotein binding proteins (PUF60, RPL36AL, ASCC3L1, MRPS17 and RPL5), three genes encoding transmembrane proteins (RTN1, IGSF8, CD9) and two genes associated with GTPase signaling activities (AIG1, RABL5) (Figure 4B). Analysis of the association network of identified genes (Supplementary Figure S1) suggests potential mechanisms of activation of the expression of androgen-responsive genes by DYDC2, RTN1 and C14orf147.

RTN1 (reticulon 1 or neuroendocrine specific protein), knockdown of which enables androgen-independent proliferation of LNCaP cells, is a neuroendocrine cell specific protein, localized in endoplasmic reticulum [69]. RTN1 was shown to interact with HDAC8 [70], a multifunctional histone deacetylase with dual nuclear and cytoplasmic localization [71]. Knockdown of RTN1 could increase the nuclear pool of HDAC8. Nuclear HDAC8 was shown to regulate activities of transcription factors, including nuclear hormone receptors [72]. C14orf147 (serine palmitoyltransferase, Small Subunit A, SPTSSA) is a regulatory subunit of serine palmitoyltransferase (SPT) [73]. SPT (localized in endoplasmic reticulum) is the first enzyme of the ER-localized ceramide biosynthesis pathway [74]. Ceramide was shown to inhibit AR activity and androgen independent growth of PCa cells through activation of protein phosphatase 2A (PP2A) [75]. Depletion of SPTSSA inhibits activity of SPT [76] and decreases ceramide accumulation [77], which in turn may cause partial inhibition of PP2A activity followed by AR activation. There is very little information about the function and activity of DPY30 Domain-Containing Protein 2 (DYDC2). The interaction network (Supplementary Figure S1) suggests that DYDC2 may influence AR activity through interaction with NME5 (NME/NM23 Family Member 5) or through HAT1 (histone acetyl transferase) activity.

We performed a more extensive characterization of IGSF8, the knockdown of which not only activates endogenous AR targets but also enables AI growth of LNCaP cells. The effect of IGSF8 knockdown was inhibited by the androgen antagonist Enzalutamide, indicating that it was mediated by AR. A close association of IGSF8 with androgen signaling is also indicated by a very high overlap between the sets of the genes that are affected either by the addition of androgen or by IGSF8 knockdown. IGSF8, an immunoglobulin family member, was originally identified as a binding partner of tetraspanin family proteins, CD9 and CD81, which modulates their activities [78]. There is evidence that IGSF8 may affect membrane interaction and localization of tetraspanins [12, 79]. shRNAs targeting both CD9 and CD81 were enriched in our shRNA library selection. Knockdown of CD9 with individual siRNAs inhibited the expression of all tested AR-responsive genes (Figure 3A) but did not affect AI growth of LNCaP cells (data not shown). Based on our results showing that knockdown of CD9 inhibits the expression of AR-responsive genes, we hypothesize that tetraspanins are involved in the activation of ligand-independent activity of AR. There are several pathways downstream of CD9 which, when activated, could be involved in the regulation of AR activity. CD9 activates MAP kinase pathways [80–82], which in turn may lead to AR activation by MAPK phosphorylation [83–85]. Additionally, AR and CD9 share a common binding partner, ADAM10 that has a dual function as a metalloproteinase and a co-activator of AR dependent transactivation [86, 87]. Modulation of CD9 activity through IGSF8 knockdown could lead to CD9 relocalization from membrane to the nucleus and interaction with AR. Finally, other as yet unknown pathways could be involved in CD9 dependent regulation of AR activity due to the pleotropic effects of tetraspanins. The latter hypothesis is supported by recent results [88] showing nuclear localization of both CD9 and IGSF8 in breast carcinoma cells. That study showed that nuclear CD9 may be involved in regulation of mitosis, therefore IGSF8 inhibition may activate CD9 effects on both cell division and AR transcriptional regulation.

Expression level and outcome of CD9 activity in human tumors depends on the tumor origin. CD9 expression is correlated with poor prognosis of gastric cancer whereas the expression in melanomas, myelomas, and head and neck squamous carcinomas is associated with early stages of cancer progression and positive treatment outcome [89, 90]. CD9 expression declined significantly as benign prostate epithelial cells progress from early malignancy to metastasis [91]. Recent findings suggest that the downregulation of CD9 expression is concomitant with the production of a truncated CD9 isoform through cancer specific gene fusion [92]. Moreover, the novel CD9 fusion genes in prostate cancer lack the IGSF8 binding epitope [93]. Thus it is tempting to speculate that the function of IGSF8 is linked to the suppression of androgen signaling in PCa cells expressing unaltered CD9. This is supported by evidence of IGSF8 deletions in prostate tumors. Both CD9 translocations and IGSF8 deletions are observed in 2–3% of prostate tumors. Therefore, activation of CD9 either by IGSF8 deletion or CD9 translocation could be involved in the development of CRPC.

Tumor suppressor activities of IGSF8 have been previously addressed in PCa, although with respect to cell motility rather than AR signaling [94], and in glioblastomas [95]. The involvement of CD9 and IGSF8 in glioma progression is consistent with our findings in PCa: elevation of CD9 expression and downregulation of IGSF8 correlated with progression of malignant glioma [95, 96], Although IGSF8 is frequently downregulated in gliomas, cBioPortal does not show any rearrangements of IGSF8 in gliomas. It seems likely that, in spite of the rarity of IGSF8 deletion in prostate cancers, the level of IGSF8 protein may also be reduced in prostate tumors by epigenetic mechanisms.

Known mechanisms of transition from AD to AI growth in PCa include AR overexpression, AR mutations and expression of AR splice variants. There are, however, many examples of PCa cell lines or tumors with elevated AR signaling but without obvious changes in AR itself. Our results provide, to the best of our knowledge, the first examples of the transactivation of AR signaling and the acquisition of AI growth through the inhibition of specific genes. Other trans-repressors and trans-activators of AR signaling that can confer androgen independence can be identified through the approach developed in the present study. Analysis of such regulatory genes in clinical CRPC should greatly enrich our understanding of the mechanisms of androgen independence in prostate cancer and suggest new approaches to overcoming this major clinical problem.

## MATERIALS AND METHODS

### Plasmids and vectors

Vectors containing rat probasin promoter (ARR(2)PB) [3] were obtained from Dr. Robert J. Matusik (Vanderbilt University, Nashville, TN). The promoter was inserted into DsRed and luciferase reporter lentiviral vectors to create pLR-PRBp-DsRed-DR and pLLRM-PRbp-Luc constructs correspondently (see construction details in Supplementary Methods). pLLCEmGFP lentiviral vector was previously described [97].

### Lentiviral infection

Lentiviral transduction was done as described [4] using pCMV-Δ8.9 and pVSV-G packaging constructs. Vector plasmid, pCMV- Δ8.9, and pVSV-G DNA were mixed at a 5:4:1 ratio and cotransfected into 293FT cells cultured on polyethyleneimine-coated plates [98]. Lentivirus-containing supernatants were harvested thrice, at 24, 48 and 72 hours after transfection.

### Cell culture

Human PCa cell lines LNCaP and PC3 from the ATCC (Manassas, VA) were maintained in RPMI-1640 medium with 10% fetal bovine serum (FBS) or switched to phenol red-free RPMI-1640 with 10% charcoal-stripped FBS (CS-FBS) for androgen-depleted conditions as previously described [99]. The 293FT cells from Life Technologies (Grand Island, NY) were maintained in DMEM with 10% FBS. R1881 (methyltrienolone), was obtained from PerkinElmer Life Sciences (Boston, MA). Reporter cell lines Prb-DsRed-LNCaP and Prb-Luc-LNCaP were prepared as described in Supplementary Methods.

### Construction of normalized shRNA libraries

The shRNA library originating from MCF7 breast carcinoma cDNA was previously reported [4]. Preparation of the prostate-specific shRNA library was similar to previously described procedure [4]. (See construction details in Supplementary Methods.)

### Library transduction, selection for activation of probasin promoter and analysis of selection results

shRNA libraries were transduced into Prb-DsRed-LNCaP reporter cells. The infection rate (as determined by qPCR analysis of integrated provirus) was 95%. The total of 10^8^ cells were plated at a density of 10^7^ cells per plate, and cultured in androgen-free conditions (phenol red-free RPMI-1640 with 10% CS-FBS) for 10 days. 25% of the cells were collected for DNA purification, the rest were subjected to FACS sorting to collect the total of 6×10^5^ DsRed positive cells. DNA was purified from unsorted and DsRed positive cells. The shRNA inserts were amplified by two rounds of PCR from genomic DNA preparations of the unsorted and DsRed positive cells and sequenced using 454 Sequencer (Roche). shRNA sequence attribution was done as previously described [4, 97].

### Screening of siRNAs and luciferase activity assay

Four siRNAs per gene, obtained from Qiagen Human Whole Genome siRNA set 1.0, were transfected into Prb-Luc-LNCaP cells in PEI coated 96-well plates (10, 000 cells per well) [98], in triplicate, at 5 nM of siRNA per well using Silentfect transfection reagent (BioRad) and reverse transfection procedure per Qiagen (Highperfect) instructions. A cytotoxic mixture of siRNAs derived from several essential genes (Qiagen, All-star cell death Hs siRNA, #1027298) and anti-luciferase siRNA (Qiagen, Luciferase GL3 siRNA, # 1022073), were used as positive controls, and siRNA targeting no known genes (Qiagen, Negative Control siRNA #1022076) was used as a negative control. Cells were cultured in phenol red-free RPMI-1640 with 10% CS-FBS 6 days post transfection, washed with PBS and lysed for 60 min with 100 μL of cell lysis buffer (0.22% NaCl, 0.15% Saponin, 1 mM EDTA) containing 0.5 μg/mL Hoechst 33342 (Polysciences Inc, Warrington, PA, #23491-52-3) that stains cellular DNA. Relative cell numbers were determined by Hoechst 33342 fluorescence. Luciferase activity was determined with prepared luciferase reporter reagents [100]. The ratio of firefly luciferase activity/Hoechst 33342 fluorescence was scored as normalized luciferase activity.

### Cell proliferation assay

LNCaP cells were transfected with siRNAs in PEI coated 96-well plates (2000 cells per well) as described above. Cell proliferation was analyzed by WST-1 assay with WST-1 cell proliferation reagent (Roche Lifescience, Indianapolis, IN).

### QPCR and microarray analysis of gene expression

LNCaP cells were transfected with siRNAs against indicated genes (Supplementary Table S7) as described above. Total cellular RNA was purified with either RNeasy kit (Qiagen) or TRIzol (Life Technologies, Grand Island, NY). Microarray analysis using Affymetrix Exon 1.0ST human oligonucleotide arrays was conducted by Ordway Research Institute's Microarray Facility. Microarray data were analyzed using GeneSpring GX (Agilent, Santa Clara CA). For quantitative PCR (qPCR) analysis, cDNA was prepared using Maxima First Strand cDNA Synthesis (Thermo Scientific, Waltham, MA). Gene expression was measured by QPCR, with gene specific primers and GAPDH or RPL13A as normalization standards (primer sequences are presented in Supplementary Table S8) using RT2 SYBR Green qPCR Master Mixes (Qiagen).

#### Protein measurement

Protein levels were assayed by immunoblotting with appropriate antibodies. The following antibodies were used: rabbit anti AR (, H-280, sc-13062, Santa Cruz, CA), rabbit anti PSA (K92110R, BioDesign Saint-Priest, France) and mouse anti GAPDH (6C5, sc-32233, Santa Cruz, CA). Appropriate secondary antibodies conjugated to horseradish peroxidase were used, and membranes were developed using an enhanced chemilluminescence reagent (Thermo Fisher Scientific Inc., Rockford, IL). Images were obtained with ChemDoc Touch imaging system (Bio-Rad, Hercules, CA). Band intensities were measured using Image Lab (Bio-Rad, Hercules, CA).

#### Immunofluorescence

Cells cultured on glass coverslips (Bellco Glass, Vineland, NJ) were fixed with 4% paraformaldehyde in PBS for 20 minutes at room temperature and permeabilized with 0.5% Triton X-100 in PBS for 3 minutes. The coverslips were incubated with rabbit anti-AR H-280 antibodies and DAPI (Life Technologies, CA). The secondary antibody was labeled with Alexa Fluor 488 (Life Technologies, CA). Fluorescence images were acquired with a 40x/0.60 n.a. LUCPlan FLN objective on an Olympus IX81 microscope. Hamamatsu C10600 camera gain and exposure time settings were controlled with Metamorph Basic. Nuclear fluorescence intensities were measured with ImageJ (NIH).

## SUPPLEMENTARY FIGURES AND TABLES




