# Supplementary material for: Identification of novel genes that regulate androgen receptor signaling and growth of androgen-deprived prostate cancer cells
Source: Oncotarget. 2015 Apr 23;6(15):13088–104. doi: 10.18632/oncotarget.3743 (PMC4537001; doi:10.18632/oncotarget.3743)
Supplement: Supplementary file 1 [file oncotarget-06-13088-s001.pdf]

SUPPLEMENTARY METHODS

Construction of reporter vectors

To create a lentiviral fluorescent reporter vector pLR-PRBp-DsRed-DR, the probasin promoter and DsRed-DR were amplified by PCR with PRB2-S-Nsi (ATTCATGCATGACGGTATCGATAAGCTTGG) and DsR-AS-st-Sph (ATTCGCAT GCCTAGGCGCG CTCGTACTGCTCCACG) and cloned into pLLRM lentiviral vector 77 using NsiI and SphI restriction sites. To create firefly luciferase based reporter vector, pLLRM-PRbp-Luc, rat probasin promoter was re-cloned from pLR-PRBp-DsRed-DR to pLLRM-FF-luc vector using PspOMI and Age I restriction sites. All enzymes were obtained from NEB (Ipswich, MA) or Thermo Scientific (Waltham, MA).

Construction of normalized shRNA library

cDNAs from RNA of LNCaP and PC3 cells were prepared with SMART cDNA synthesis kit (Clontech Laboratories, Mountain View, CA). cDNA preparations were mixed and normalized by Evrogen (Moscow, Russia) using duplex-specific nuclease 101. Normalized cDNA mixtures were fragmented as described[1] followed by ligation with hairpin adapter (Mme-loop-AD), containing MmeI restriction site. Fragments were digested with MmeI (NEB, Ipswich, MA); the resulting pool of ~40 bp fragments was gel purified, ligated with termination adapter (TA), followed by extension with Klenow fragment of DNA polymerase I. 139-bp to 143-bp long extended fragments were further purified on an 8% TBE-polyacrylamide gel, digested with MlyI and XbaI restriction enzymes and inserted into LLCEP TU6LX lentiviral vector. The resulting library containing ~1.5 million independent clones was transformed into 0G Supreme E. Coli 1 (Lucigen, Middleton, WI). 100 clones were randomly verified by Sanger sequencing followed by analysis of insert integrity. The results showed that 92% of the clones contained correct shRNA inserts.

Preparation of reporter cell lines

The reporter cell line, Prb-DsRed-LNCaP was created by infection of LNCaP cells with pLR-PRBp-DsRed-DR lentivirus followed by treatment with 100 pM of R1881 for 48 h and FACS sorting of DsRed positive cells. The sorted population was replated and, after recovery, cultured for 72 h in androgen-depleted conditions, followed by sorting of DsRed negative cells. DsRed positive and negative cells were subjected to the sorting procedure twice to ensure minimal background of DsRed fluorescence. Reporter cell line Prb-Luc-LNCaP was created by infection of LNCaP cells with pLR-PRBp-FF luc lentiviral vector at MOI ~2. MOI was determined by qPCR with lentivirus-specific primers.

1. Primiano T, Baig M, Maliyekkel A, Chang BD, Fellars S, Sadhu J, et al. Identification of potential anticancer drug targets through the selection of growth-inhibitory genetic suppressor elements. Cancer Cell. 2003; 4:41–53.

REFERENCES

1. Sharma, M., Sun, Z. (2001) Mol. Endocrinol. 15, 1918–1928.
2. Zhang, Y., Akinmade, D., Hamburger, A. W. (2005) Nucleic Acids Res. 33, 6024–6033.
3. Powzaniuk, M., McElwee-Witmer, S., Vogel, R. L., Hayami, T., Rutledge, S. J., Chen, F., Harada, S., Schmidt, A., Rodan, G. A., Freedman, L. P. et al. (2004) Mol. Endocrinol. 18, 2011–2023.
4. Cao, X., Qin, J., Xie, Y., Khan, O., Dowd, F., Scofield, M., Lin, M. F., Tu, Y. (2006) Oncogene 25, 3719–3734.
5. Lee, Y. F., Shyr, C. R., Thin, T. H., Lin, W. J., Chang, C. (1999) Proc. Natl. Acad. Sci. U. S. A 96, 14724–14729.
6. Fan, M., Long, X., Bailey, J. A., Reed, C. A., Osborne, E., Gize, E. A., Kirk, E. A., Bigsby, R. M., Nephew, K. P. (2002) Mol. Endocrinol. 16, 315–330.
7. Meehan, K. L., Holland, J. W., Dawkins, H. J. (2002) Prostate 50, 54–63.
8. Norris, J. D., Chang, C. Y., Wittmann, B. M., Kunder, R. S., Cui, H., Fan, D., Joseph, J. D., McDonnell, D. P. (2009) Mol. Cell 36, 405–416.
9. Jung, C., Kim, R. S., Zhang, H. J., Lee, S. J., Jeng, M. H. (2004) Cancer Res. 64, 9185–9192.
10. Schulz, W. A., Alexa, A., Jung, V., Hader, C., Hoffmann, M. J., Yamanaka, M., Fritzsche, S., Wlazlinski, A., Muller, M., Lengauer, T. et al. (2007) Mol. Cancer 6, 14.
11. Callewaert, L., Verrijdt, G., Haelens, A., Claessens, F. (2004) Mol. Endocrinol. 18, 1438–1449.
12. Kaikkonen, S., Jaaskelainen, T., Karvonen, U., Rytinki, M. M., Makkonen, H., Gioeli, D., Paschal, B. M., Palvimo, J. J. (2009) Mol. Endocrinol. 23, 292–307.
13. Hisatake, J. I., Ikezoe, T., Carey, M., Holden, S., Tomoyasu, S., Koeffler, H. P. (2000) Cancer Res. 60, 5494–5498.
14. Nakamura, Y., Suzuki, T., Sugawara, A., Arai, Y., Sasano, H. (2009) Pathol. Int. 59, 288–293.
15. Pero, R., Lembo, F., Palmieri, E. A., Vitiello, C., Fedele, M., Fusco, A., Bruni, C. B., Chiariotti, L. (2002) J. Biol. Chem. 277, 3280–3285.
16. Takahara, K., Azuma, H., Sakamoto, T., Kiyama, S., Inamoto, T., Ibuki, N., Nishida, T., Nomi, H., Ubai, T., Segawa, N. et al. (2009) Anticancer Res. 29, 2497–2505.
17. Juarranz, M. G., Bolanos, O., Gutierrez-Canas, I., Lerner, E. A., Robberecht, P., Carmena, M. J., Prieto, J. C., Rodriguez-Henche, N. (2001) Cell Signal. 13, 887–894.
18. Mukhopadhyay, N. K., Cinar, B., Mukhopadhyay, L., Lutchman, M., Ferdinand, A. S., Kim, J., Chung, L. W., Adam, R. M., Ray, S. K., Leiter, A. B. et al. (2007) Mol. Endocrinol. 21, 2056–2070.
19. Nuber, U. A., Kriaucionis, S., Roloff, T. C., Guy, J., Selfridge, J., Steinhoff, C., Schulz, R., Lipkowitz, B., Ropers, H. H., Holmes, M. C. et al. (2005) Hum. Mol. Genet. 14, 2247–2256.
20. Sharifi, N., Hurt, E. M., Thomas, S. B., Farrar, W. L. (2008) Clin. Cancer Res. 14, 6073–6080.
21. Chan, J. M., Oh, W. K., Xie, W., Regan, M. M., Stampfer, M. J., King, I. B., Abe, M., Kantoff, P. W. (2009) J. Clin. Oncol. 27, 3577–3583.
22. Suh, J. H., Gong, E. Y., Kim, J. B., Lee, I. K., Choi, H. S., Lee, K. (2008) Mol. Cancer Res. 6, 314–324.
23. Fan, M., Long, X., Bailey, J. A., Reed, C. A., Osborne, E., Gize, E. A., Kirk, E. A., Bigsby, R. M., Nephew, K. P. (2002) Mol. Endocrinol. 16, 315–330.
24. Jeong, B. C., Hong, C. Y., Chattopadhyay, S., Park, J. H., Gong, E. Y., Kim, H. J., Chun, S. Y., Lee, K. (2004) Mol. Endocrinol. 18, 13–25.
25. Wang, Y., Li, T., Qiu, X., Mo, X., Zhang, Y., Song, Q., Ma, D., Han, W. (2008) Biochem. Biophys. Res. Commun. 371, 54–58.
26. Davis, J. N., Wojno, K. J., Daignault, S., Hofer, M. D., Kuefer, R., Rubin, M. A., Day, M. L. (2006) Cancer Res. 66, 11897–11906.
27. Cai, C., Portnoy, D. C., Wang, H., Jiang, X., Chen, S., Balk, S. P. (2009) Cancer Res. 69, 5202–5209.
28. Yu, X., Li, P., Roeder, R. G., Wang, Z. (2001) Mol. Cell Biol. 21, 4614–4625.
29. Lin, D. Y., Fang, H. I., Ma, A. H., Huang, Y. S., Pu, Y. S., Jenster, G., Kung, H. J., Shih, H. M. (2004) Mol. Cell Biol. 24, 10529–10541.
30. Sharma, M., Sun, Z. (2001) Mol. Endocrinol. 15, 1918–1928.
31. Isayeva, T., Moore, L. D., Chanda, D., Chen, D., Ponnazhagan, S. (2009) Prostate 69, 1055–1066.
32. Isayeva, T., Moore, L. D., Chanda, D., Chen, D., Ponnazhagan, S. (2009) Prostate 69, 1055–1066.
33. Chouinard, S., Barbier, O., Belanger, A. (2007) J. Biol. Chem. 282, 33466–33474.
34. Park, J., Chen, L., Ratnashinge, L., Sellers, T. A., Tanner, J. P., Lee, J. H., Dossett, N., Lang, N., Kadlubar, F. F., Ambrosone, C. B. et al. (2006) Cancer Epidemiol. Biomarkers Prev. 15, 1473–1478.
35. Zheng, Z., Cai, C., Omwancha, J., Chen, S. Y., Baslan, T., Shemshedini, L. (2006) J. Biol. Chem. 281, 4002–4012.
36. Bigler, D., Gioeli, D., Conaway, M. R., Weber, M. J., Theodorescu, D. (2007) Prostate 67, 1590–1599.
37. Kawate, H., Wu, Y., Ohnaka, K., Takayanagi, R. (2007) J. Steroid Biochem. Mol. Biol. 105, 46–56.
38. Brown, D. A., Scholfield, C. N. (1984) Br. J. Pharmacol. 83, 195–202.
39. Brubaker, K. D., Vessella, R. L., Brown, L. G., Corey, E. (2003) Prostate 56, 13–22.

40. Zaidi, S. K., Pande, S., Pratap, J., Gaur, T., Grigoriu, S., Ali, S. A., Stein, J. L., Lian, J. B., van Wijnen, A. J., Stein, G. S. (2007) Proc. Natl. Acad. Sci. U. S. A 104, 19861–19866.
41. Jana, N. R., Sarkar, S., Ishizuka, M., Yonemoto, J., Tohyama, C., Sone, H. (1999) Biochem. Biophys. Res. Commun. 256, 462–468.
42. Morrow, D., Qin, C., Smith, R., III, Safe, S. (2004) J. Steroid Biochem. Mol. Biol. 88, 27–36.
43. Kashani, M., Steiner, G., Haitel, A., Schaufler, K., Thalhammer, T., Amann, G., Kramer, G., Marberger, M., Scholler, A. (1998) Prostate 37, 98–108.
44. Liu, P., Li, S., Gan, L., Kao, T. P., Huang, H. (2008) Cancer Res. 68, 10290–10299.
45. Welsbie, D. S., Xu, J., Chen, Y., Borsu, L., Scher, H. I., Rosen, N., Sawyers, C. L. (2009) Cancer Res. 69, 958–966.
46. Geserick, C., Meyer, H. A., Barbulescu, K., Haendler, B. (2003) Mol. Endocrinol. 17, 1738–1750.
47. Carsol, J. L., Gingras, S., Simard, J. (2002) Mol. Endocrinol. 16, 1696–1710.
48. Park, J. J., Irvine, R. A., Buchanan, G., Koh, S. S., Park, J. M., Tilley, W. D., Stallcup, M. R., Press, M. F., Coetzee, G. A. (2000) Cancer Res. 60, 5946–5949.
49. Gaughan, L., Logan, I. R., Cook, S., Neal, D. E., Robson, C. N. (2002) J. Biol. Chem. 277, 25904–25913.
50. Niki, T., Takahashi-Niki, K., Taira, T., Iguchi-Ariga, S. M., Ariga, H. (2003) Mol. Cancer Res. 1, 247–261.
51. Welsbie, D. S., Xu, J., Chen, Y., Borsu, L., Scher, H. I., Rosen, N., Sawyers, C. L. (2009) Cancer Res. 69, 958–966.
52. Sharma, M., Li, X., Wang, Y., Zarnegar, M., Huang, C. Y., Palvimo, J. J., Lim, B., Sun, Z. (2003) EMBO J. 22, 6101–6114.
53. Vlahopoulos, S., Zimmer, W. E., Jenster, G., Belaguli, N. S., Balk, S. P., Brinkmann, A. O., Lanz, R. B., Zoumpourlis, V. C., Schwartz, R. J. (2005) J. Biol. Chem. 280, 7786–7792.
54. Heemers, H. V., Regan, K. M., Dehm, S. M., Tindall, D. J. (2007) Cancer Res. 67, 10592–10599.
55. Heemers, H. V., Regan, K. M., Dehm, S. M., Tindall, D. J. (2007) Cancer Res. 67, 10592–10599.
56. Link, K. A., Burd, C. J., Williams, E., Marshall, T., Rosson, G., Henry, E., Weissman, B., Knudsen, K. E. (2005) Mol. Cell Biol. 25, 2200–2215.
57. Link, K. A., Balasubramaniam, S., Sharma, A., Comstock, C. E., Godoy-Tundidor, S., Powers, N., Cao, K. H., Haelens, A., Claessens, F., Revelo, M. P. et al. (2008) Cancer Res. 68, 4551–4558.
58. Link, K. A., Balasubramaniam, S., Sharma, A., Comstock, C. E., Godoy-Tundidor, S., Powers, N., Cao, K. H., Haelens, A., Claessens, F., Revelo, M. P. et al. (2008) Cancer Res. 68, 4551–4558.
59. Chen, S., Xu, Y., Yuan, X., Bubley, G. J., Balk, S. P. (2006) Proc. Natl. Acad. Sci. U. S. A 103, 15969–15974.
60. Hosohata, K., Li, P., Hosohata, Y., Qin, J., Roeder, R. G., Wang, Z. (2003) Mol. Cell Biol. 23, 7019–7029.
61. Kang, N., Duan, L., Tang, L., Liu, S., Li, C., Li, Y., Liu, Q., Hu, Y., Cui, L., He, W. (2008) Cell Mol. Immunol. 5, 279–285.
62. Yeh, S., Chang, C. (1996) Proc. Natl. Acad. Sci. U. S. A 93, 5517–5521.
63. Culig, Z., Comuzzi, B., Steiner, H., Bartsch, G., Hobisch, A. (2004) J. Steroid Biochem. Mol. Biol. 92, 265–271.
64. Stanbrough, M., Bubley, G. J., Ross, K., Golub, T. R., Rubin, M. A., Penning, T. M., Febbo, P. G., Balk, S. P. (2006) Cancer Res. 66, 2815–2825.
65. Stanbrough, M., Bubley, G. J., Ross, K., Golub, T. R., Rubin, M. A., Penning, T. M., Febbo, P. G., Balk, S. P. (2006) Cancer Res. 66, 2815–2825.
66. Hosohata, K., Li, P., Hosohata, Y., Qin, J., Roeder, R. G., Wang, Z. (2003) Mol. Cell Biol. 23, 7019–7029.
67. Shi, Y. K., Yu, Y. P., Zhu, Z. H., Han, Y. C., Ren, B., Nelson, J. B., Luo, J. H. (2008) Am. J. Pathol. 173, 1758–1767.
68. Laitinen, S., Martikainen, P. M., Tolonen, T., Isola, J., Tammela, T. L., Visakorpi, T. (2008) Int. J. Cancer 122, 595–602.
69. Wolf, S. S., Patchev, V. K., Obendorf, M. (2007) Arch. Biochem. Biophys. 460, 56–66.
70. Dos Santos Neto, J. G. (1975) Am. J. Clin. Pathol. 63, 909–915.
71. Hsiao, P. W., Chang, C. (1999) J. Biol. Chem. 274, 22373–22379.
72. Abrham, G., Volpe, M., Shpungin, S., Nir, U. (2009) Int. J. Cancer 125, 43–53.
73. Tan, S. H., Dagvadorj, A., Shen, F., Gu, L., Liao, Z., Abdulghani, J., Zhang, Y., Gelmann, E. P., Zellweger, T., Culig, Z. et al. (2008) Cancer Res. 68, 236–248.
74. Tan, S. H., Nevalainen, M. T. (2008) Endocr. Relat Cancer 15, 367–390.
75. Nair, S. S., Guo, Z., Mueller, J. M., Koochekpour, S., Qiu, Y., Tekmal, R. R., Schule, R., Kung, H. J., Kumar, R., Vadlamudi, R. K. (2007) Mol. Endocrinol. 21, 613–624.
76. Fu, M., Rao, M., Wang, C., Sakamaki, T., Wang, J., Di Vizio, D., Zhang, X., Albanese, C., Balk, S., Chang, C. et al. (2003) Mol. Cell Biol. 23, 8563–8575.
77. Zhao, Y., Lang, G., Ito, S., Bonnet, J., Metzger, E., Sawatsubashi, S., Suzuki, E., Le, G., X, Stunnenberg, H. G., Krasnov, A. et al. (2008) Mol. Cell 29, 92–101.

78. Harada, N., Ohmori, Y., Yamaji, R., Higashimura, Y., Okamoto, K., Isohashi, F., Nakano, Y., Inui, H. (2008) Biochem. Biophys. Res. Commun. 373, 373–377.
79. Metzger, E., Wissmann, M., Yin, N., Muller, J. M., Schneider, R., Peters, A. H., Gunther, T., Buettner, R., Schule, R. (2005) Nature 437, 436–439.
80. Kahl, P., Gullotti, L., Heukamp, L. C., Wolf, S., Friedrichs, N., Vorreuther, R., Solleder, G., Bastian, P. J., Ellinger, J., Metzger, E. et al. (2006) Cancer Res. 66, 11341–11347.
81. Ting, H. J., Yeh, S., Nishimura, K., Chang, C. (2002) Proc. Natl. Acad. Sci. U. S. A 99, 661–666.
82. Chen, D., Ma, H., Hong, H., Koh, S. S., Huang, S. M., Schurter, B. T., Aswad, D. W., Stallcup, M. R. (1999) Science 284, 2174–2177.
83. Zou, J. X., Guo, L., Revenko, A. S., Tepper, C. G., Gemo, A. T., Kung, H. J., Chen, H. W. (2009) Cancer Res. 69, 3339–3346.
84. Takahashi, K., Taira, T., Niki, T., Seino, C., Iguchi-Ariga, S. M., Ariga, H. (2001) J. Biol. Chem. 276, 37556–37563.
85. Leygue, E. (2007) Nucl. Recept. Signal. 5, e006.
86. Kang, H. B., Choi, Y., Lee, J. M., Choi, K. C., Kim, H. C., Yoo, J. Y., Lee, Y. H., Yoon, H. G. (2009) FEBS Lett. 583, 1880–1886.
87. Khan, O. Y., Fu, G., Ismail, A., Srinivasan, S., Cao, X., Tu, Y., Lu, S., Nawaz, Z. (2006) Mol. Endocrinol. 20, 544–559.
88. Truica, C. I., Byers, S., Gelmann, E. P. (2000) Cancer Res. 60, 4709–4713.
89. Elo, J. P., Visakorpi, T. (2001) Ann. Med. 33, 130–141.
90. Lin, H. K., Altuwaijri, S., Lin, W. J., Kan, P. Y., Collins, L. L., Chang, C. (2002) J. Biol. Chem. 277, 36570–36576.
91. Lin, H. K., Altuwaijri, S., Lin, W. J., Kan, P. Y., Collins, L. L., Chang, C. (2002) J. Biol. Chem. 277, 36570–36576.
92. Lin, H. K., Altuwaijri, S., Lin, W. J., Kan, P. Y., Collins, L. L., Chang, C. (2002) J. Biol. Chem. 277, 36570–36576.
93. Lin, H. K., Altuwaijri, S., Lin, W. J., Kan, P. Y., Collins, L. L., Chang, C. (2002) J. Biol. Chem. 277, 36570–36576.
94. Hong, C. Y., Suh, J. H., Kim, K., Gong, E. Y., Jeon, S. H., Ko, M., Seong, R. H., Kwon, H. B., Lee, K. (2005) Mol. Cell Biol. 25, 4841–4852.
95. Heeboll, S., Borre, M., Ottosen, P. D., Andersen, C. L., Mansilla, F., Dyrskjot, L., Orntoft, T. F., Torring, N. (2008) Histol. Histopathol. 23, 1069–1076.
96. Wissmann, M., Yin, N., Muller, J. M., Greschik, H., Fodor, B. D., Jenuwein, T., Vogler, C., Schneider, R., Gunther, T., Buettner, R. et al. (2007) Nat. Cell Biol. 9, 347–353.
97. Sharma, M., Li, X., Wang, Y., Zarnegar, M., Huang, C. Y., Palvimo, J. J., Lim, B., Sun, Z. (2003) EMBO J. 22, 6101–6114.
98. Wafa, L. A., Palmer, J., Fazli, L., Hurtado-Coll, A., Bell, R. H., Nelson, C. C., Gleave, M. E., Cox, M. E., Rennie, P. S. (2007) Hum. Pathol. 38, 161–170.
99. Zhao, Y., Lang, G., Ito, S., Bonnet, J., Metzger, E., Sawatsubashi, S., Suzuki, E., Le, G., X, Stunnenberg, H. G., Krasnov, A. et al. (2008) Mol. Cell 29, 92–101.

SUPPLEMENTARY FIGURES AND TABLES

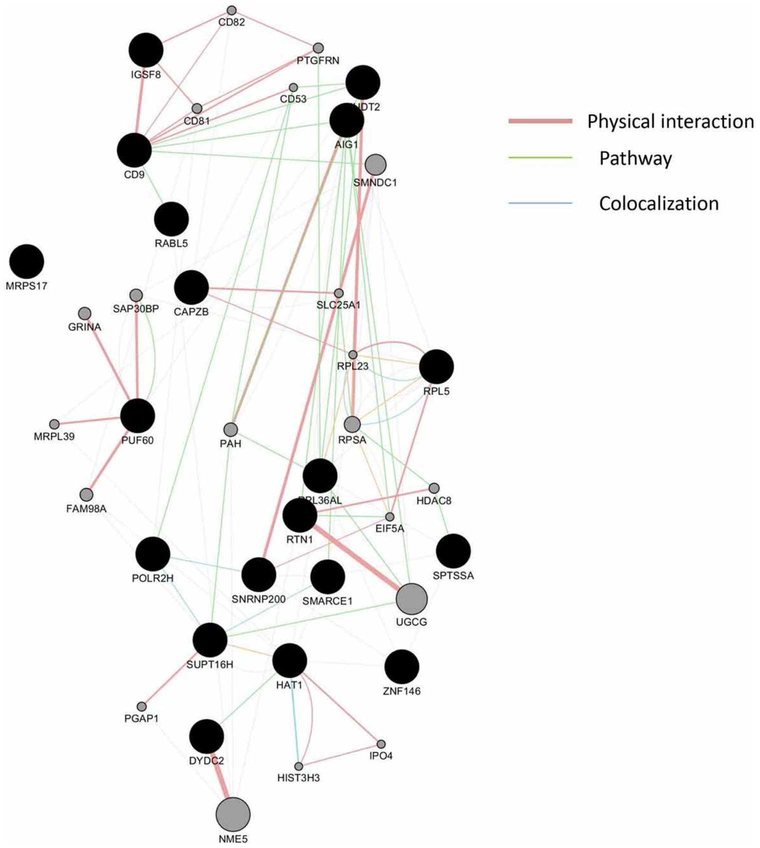

Supplementary Figure S1: Interaction network of new regulators of androgen responsive genes found in functional screening. Network of physical and functional interactions of identified genes was constructed using Cytoscape geneMANIA plugin. Black nodes represents query (screen identified) genes, grey nodes represent additional genes, predicted to be related to the network.

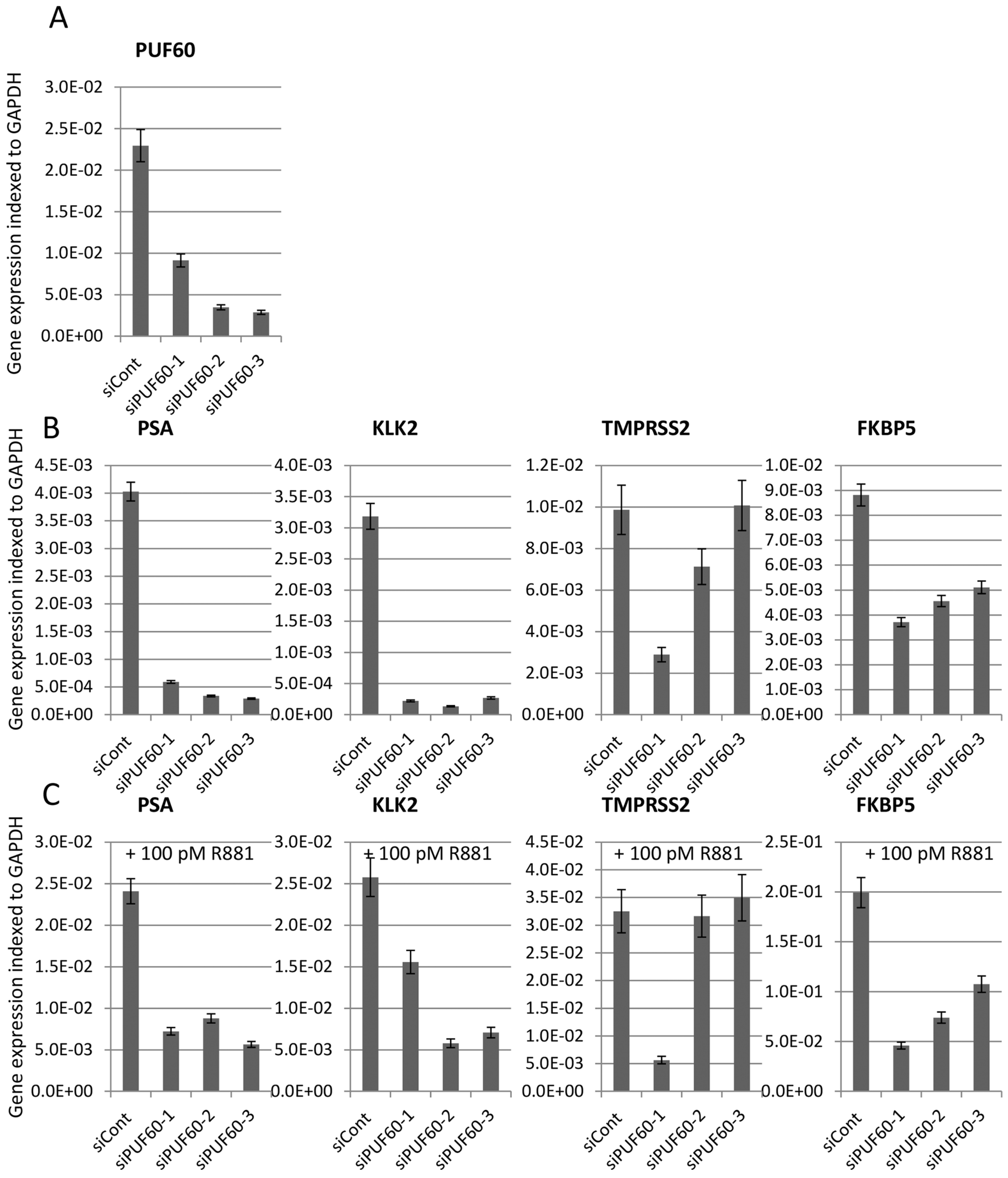

Supplementary Figure S2: siRNA PUF60 antagonizes AR activity both in activation and repression of target genes transcription. A. Expression of PUF60 gene in LNCaP cells transfected with different PUF60 targeting siRNAs **B–C.** Expression of endogenous genes positively regulated by AR (PSA, KLK2, TMPRSS2 and FKBP5) is repressed by PUF60 knockdown both in the presence **D.** or absence **C.** of androgen. LNCaP cells were transfected with control or each of three different siRNAs against PUF60 (5 nM) in triplicates. Cells were cultured in hormone-free conditions for 6 days followed by treatment with either 100 pM of R1881 or vehicle control for 24 h. Expression of indicated genes was analyzed by QPCR. Results represent mean of 3 independent experiments +/– SD.

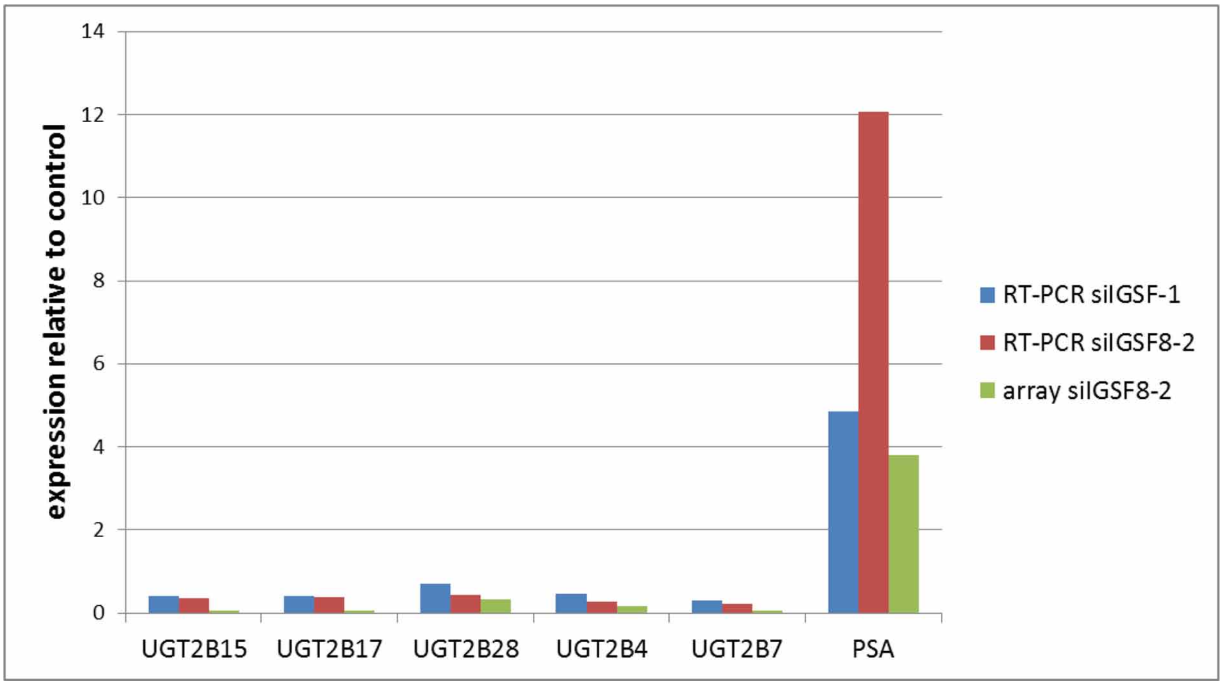

Supplementary Figure S3: Knocking down components of the IGSF8 inhibited expression of UDP glucuronosyltransferase 2 family genes. RT-PCR and microarray results of UDP glucuronosyltransferase 2 family gene expression in LNCaP cells transfected with siRNAs targeting IGSF8. Results represent fold expression relative to control (LNCaP cells transfected with Qiagen, Negative Control siRNA).

Supplementary Table S1: Analysis of selections of shRNA libraries

	MCF-7 shRNA libary		Prostate specific shRNA libary	
	Infected set	FACS selected set	Infected set	FACS selected set
Total number of reads	51618	43777	61410	49503
Number of meaningful reads	31199	25311	21259	15458
Number of genes targeted by reads	11032	4463	4558	3509
Number of genes with 4 or more fold enrichment in selected subsets		1451		1462

Supplementary Table S2: Previously described regulators of activity of androgen receptor revealed in selections of shRNA libraries

Inhibitors of AR transcriptional activity

Gene Symbol	Gene Name	Relative enrichment	Regulation of AR activity	Involvement in prostate cancer progression
SIN3A	SIN3 homolog A, transcription regulator	63.21	(1, 2)	
LATS2	LATS, large tumor suppressor, homolog 2	39.51	(3)	
RGS2	Regulator of G-protein signalling 2, 24kDa	38.84	(4)	
NR2C2	Nuclear receptor subfamily 2, group C, member 2	38.84	(5)	
NEDD8	Neural precursor cell expressed, developmentally down-regulated 8	38.84	(6)	(7)
HOXB13	Homeobox B13	25.89	(8, 9)	(10)
SUMO1	SMT3 suppressor of mif two 3 homolog 1	12.95	(11, 12)	
PPARG	Peroxisome proliferator-activated receptor gamma	12.95	(13)	(14)
PATZ1	POZ (BTB) and AT hook containing zinc finger 1	12.95	(15)	
AMACR	Alpha-methylacyl-CoA racemase	12.95	(16)	
VIPR1	Vasoactive intestinal peptide receptor 1	4.93	(17)	
RREB1	Ras responsive element binding protein 1	4.13	(18)	
MECP2	Methyl CpG binding protein 2 (Rett syndrome)	3.90	(19)	
SOD2	Superoxide dismutase 2, mitochondrial	3.70	(20)	(21)
SREBF1	Sterol regulatory element binding transcription factor 1	3.70	(22)	
UBE1C	Ubiquitin-activating enzyme E1C (UBA3 homolog, yeast)	3.39	(23)	
HDAC4	Histone deacetylase 4	2.88	(24)	
CMTM3	CKLF-like MARVEL transmembrane domain containing 3	2.75	(25)	
E2F1	E2F transcription factor 1	2.74	(26)	
ERBB2	V-erb-b2 erythroblastic leukemia viral oncogene homolog 2, neuro/glioblastoma derived oncogene homolog (avian)	2.71	(27)	
AES	Amino-terminal enhancer of split	2.62	(28)	
DAXX	Death-associated protein 6	2.47	(29)	

(*Continued*)

Gene Symbol	Gene Name	Relative enrichment	Regulation of AR activity	Involvement in prostate cancer progression
TGIF1	TGFB-induced factor homeobox 1	2.47	(30)	
COL18A1	Collagen, type XVIII, alpha 1	2.00	(31)	(32)
UGT2B17	UDP glucuronosyltransferase 2 family, polypeptide B17	2.75	(33)	(34)

Dual (Negative and Positive) regulators of AR dependent transactivation

Gene Symbol	Gene Name	Relative enrichment	Regulation of AR activity	Involvement in prostate cancer progression
SUMO2	SMT3 suppressor of mif two 3 homolog 2 (S. cerevisiae)	25.89	(35)	
RAP2A	RAP2A, member of RAS oncogene family	15.80	(36)	
RUNX2	Runt-related transcription factor 2	12.95	(37, 38)	(39, 40)
AHR	Aryl hydrocarbon receptor	12.95	(41)	(42, 43)
HDAC3	Histone deacetylase 3	4.01	(44, 45)	
PIAS2	Protein inhibitor of activated STAT, 2	3.08	(46)	
STAT5B	Signal transducer and activator of transcription 5B	3.70	(47)	
BRCA1	Breast cancer 1, early onset	12.95	(48)	
HDAC1	Histone deacetylase 1	2.36	(49–51)	(52)

Activators of AR transcriptional activity

Gene Symbol	Gene Name	Relative enrichment	Regulation of AR activity	Involvement in prostate cancer progression
SRF	Serum response factor (c-fos serum response element-binding transcription factor)	90.63	(53, 54)	(55)
SMARCE1	Homo sapiens SWI/SNF related, matrix associated, actin dependent regulator of chromatin, subfamily e, member 1 (SMARCE1), mRNA	64.73	(56, 57)	(58)
CDC2	Cell division cycle 2, G1 to S and G2 to M	64.73	(59)	
PRMT5	Protein arginine methyltransferase 5	51.79	(60)	
RWDD1	RWD domain containing 1	39.51	(61)	
NCOA4	Homo sapiens nuclear receptor coactivator 4 (NCOA4)	39.51	(62)	(63)
SRD5A1	Steroid-5-alpha-reductase, alpha polypeptide 1 (3-oxo-5 alpha-steroid delta 4-dehydrogenase alpha 1)	38.84	(64)	(65)

(*Continued*)

Activators of AR transcriptional activity

Gene Symbol	Gene Name	Relative enrichment	Regulation of AR activity	Involvement in prostate cancer progression
PXN	Paxillin	25.89	(66)	
MCM7	MCM7 minichromosome maintenance deficient 7 (S. cerevisiae)	25.89	(67)	(68)
JMJD1C	Jumonji domain containing 1C	25.89	(69)	
TRAM1	Translocation associated membrane protein 1	12.95	(70)	
TMF1	TATA element modulatory factor 1	12.95	(71)	(72)
STAT5A	Signal transducer and activator of transcription 5A	12.95	(73)	(74)
PELP1	Proline, glutamic acid and leucine rich protein 1	12.95	(75)	
EP300	E1A binding protein p300	12.95	(76)	
ENY2	Enhancer of yellow 2 homolog (Drosophila)	11.09	(77)	
RAN	RAN, member RAS oncogene family	8.26	(78)	
AOF2	Amine oxidase (flavin containing) domain 2	7.90	(79)	(80)
SVIL	Supervillin	7.40	(81)	
CARM1	Coactivator-associated arginine methyltransferase 1	6.57	(82)	
ATAD2	ATPase family, AAA domain containing 2	6.16	(83)	
PARK7	Parkinson disease (autosomal recessive, early onset) 7	5.51	(84)	
SRA1	Steroid receptor RNA activator 1	4.93	(85)	
WHSC1	Wolf-Hirschhorn syndrome candidate 1	4.93	(86)	
UBE3A	Ubiquitin protein ligase E3A (human papilloma virus E6-associated protein, Angelman syndrome)	4.13	(87)	
CTNNB1	Catenin (cadherin-associated protein), beta 1, 88kDa	3.18	(88)	(89)
PSMA7	Proteasome (prosome, macropain) subunit, alpha type, 7	2.88	(90)	
PSMC4	Proteasome (prosome, macropain) 26S subunit, ATPase, 4	38.84	(91)	
PSMD10	Proteasome (prosome, macropain) 26S subunit, non-ATPase, 10	38.84	(92)	
PSMD14	Proteasome (prosome, macropain) 26S subunit, non-ATPase, 14	12.95	(93)	

(*Continued*)

Activators of AR transcriptional activity

Gene Symbol	Gene Name	Relative enrichment	Regulation of AR activity	Involvement in prostate cancer progression
SMARCC1	SWI/SNF related, matrix associated, actin dependent regulator of chromatin, subfamily c, member 1	2.75	(94)	(95)
JMJD2C	Jumonji domain containing 2C	2.47	(96)	
ZMIZ1	Zinc finger, MIZ-type containing 1	2.47	(97)	
DDC	Dopa decarboxylase (aromatic L-amino acid decarboxylase)	2.16	(98)	
ATXN7L3	Ataxin 7-like 3	3.08	(99)	

Supplementary Table S3: Genes selected for siRNA based validation I

Genes enriched in MCF7 (breast cancer) library selection

Gene Symbol	Annotation	Enrichment with prostate specific shRNA library selection	Enrichment with MCF7 shRNA library selection	# counts in selected subset	# counts in infected subset
TRAPPC5	Homo sapiens trafficking protein particle complex 5 (TRAPPC5), transcript variant 3, mRNA	0	118.5255423	15	< 1
FOXD1	Forkhead box D1	0	94.8204338	12	< 1
HCST	Hematopoietic cell signal transducer	0	94.8204338	12	< 1
PHLDB1	Homo sapiens pleckstrin homology-like domain, family B, member 1(PHLDB1), transcript variant 3, mRNA	0	94.8204338	12	< 1
ST3GAL6	ST3GAL6 ST3 beta-galactoside alpha-2,3-sialyltransferase 6	0	94.8204338	12	< 1
ZNF428	Homo sapiens zinc finger protein 428 (ZNF428), mRNA	0	94.8204338	12	< 1
PAXIP1	PAX interacting (with transcription-activation domain) protein 1	0	86.91873099	11	< 1
C20orf3	Homo sapiens chromosome 20 open reading frame 3 (C20orf3), mRNA	0	79.01702817	10	< 1

(*Continued*)

Genes enriched in MCF7 (breast cancer) library selection

Gene Symbol	Annotation	Enrichment with prostate specific shRNA library selection	Enrichment with MCF7 shRNA library selection	# counts in selected subset	# counts in infected subset
MLLT11	Myeloid/lymphoid or mixed-lineage leukemia (trithorax homolog, Drosophila); translocated to, 11	0	79.01702817	10	< 1
RAB6A	RAB6A, member RAS oncogene family	0	79.01702817	10	< 1
TRMT5	TRM5 tRNA methyltransferase 5 homolog (S. cerevisiae)	0	79.01702817	10	< 1
ZNF195	Zinc finger protein 195	0.635212923	79.01702817	10	< 1
NDUFA10	NADH dehydrogenase (ubiquinone) 1 alpha subcomplex, 10, 42kDa	0	71.11532535	9	< 1
NKRF	NF-kappaB repressing factor	0	71.11532535	9	< 1
PCSK5	Proprotein convertase subtilisin/kexin type 5	0	71.11532535	9	< 1
RGNEF	Rho-guanine nucleotide exchange factor	0	71.11532535	9	< 1
ADCK5	AarF domain containing kinase 5	0	63.21362254	8	< 1
EYA3	EYA3 eyes absent homolog 3 (Drosophila) (potential alternate splice variant with extended 3’ UTR	0	63.21362254	8	< 1
KIF7	Homo sapiens kinesin family member 7 (KIF7), mRNA	0	63.21362254	8	< 1
KRT8	Homo sapiens keratin 8 (KRT8), mRNA (homologue with multiple pseudogenes)	0	63.21362254	8	< 1
LCOR	Ligand dependent nuclear receptor corepressor	0	63.21362254	8	< 1
LPIN1	Lipin 1	0	63.21362254	8	< 1
PIWIL2	Homo sapiens piwi-like 2 (Drosophila) (PIWIL2), transcript variant 1, mRNA (homology with multiple genes)	0	63.21362254	8	< 1
RAD17	RAD17 homolog (S. pombe)	0	63.21362254	8	< 1
SIN3A	SIN3 homolog A, transcription regulator (yeast)	0	63.21362254	8	< 1

(*Continued*)

Genes enriched in MCF7 (breast cancer) library selection

Gene Symbol	Annotation	Enrichment with prostate specific shRNA library selection	Enrichment with MCF7 shRNA library selection	# counts in selected subset	# counts in infected subset
ALG1	Homo sapiens asparagine-linked glycosylation 1, beta-1,4-mannosyltransferase homolog (S. cerevisiae) (ALG1), mRNA (multiple homology with other genes)	0	55.31191972	7	< 1
C16orf7	Chromosome 16 open reading frame 7	0	55.31191972	7	< 1
C9orf58	Chromosome 9 open reading frame 58	0	55.31191972	7	< 1
CAPZB	Homo sapiens capping protein (actin filament) muscle Z-line, beta (CAPZB), mRNA	0	55.31191972	7	< 1
CD9	Homo sapiens CD9 molecule (CD9), mRNA	0	55.31191972	7	< 1
COX7C	Cytochrome c oxidase subunit VIIc	2.322497249	55.31191972	7	< 1
CYP2D7P1	Cytochrome P450, family 2, subfamily D, polypeptide 7 pseudogene 1	0	55.31191972	7	< 1
EXTL1	Exostoses (multiple)-like 1	0	55.31191972	7	< 1
LCN2	Lipocalin 2 (oncogene 24p3)	0	55.31191972	7	< 1
RAB13	RAB13, member RAS oncogene family	0	55.31191972	7	< 1
REEP1	Receptor accessory protein 1	0	55.31191972	7	< 1
SCOTIN	Homo sapiens shisa homolog 5 (Xenopus laevis) (SHISA5), mRNA	1.376294666	55.31191972	7	< 1
SMARCA5	SWI/SNF related, matrix associated, actin dependent regulator of chromatin, subfamily a, member 5	0	55.31191972	7	< 1
SNRPF	Small nuclear ribonucleoprotein polypeptide F	1.376294666	55.31191972	7	< 1
ZNF23	Zinc finger protein 23 (KOX 16)	0	55.31191972	7	< 1
ZNF367	Zinc finger protein 367	0	55.31191972	7	< 1
ADCY3	Homo sapiens adenylate cyclase 3 (ADCY3), mRNA	0	47.4102169	6	< 1

(*Continued*)

Genes enriched in MCF7 (breast cancer) library selection

Gene Symbol	Annotation	Enrichment with prostate specific shRNA library selection	Enrichment with MCF7 shRNA library selection	# counts in selected subset	# counts in infected subset
AHCTF1	AT hook containing transcription factor 1	0	47.4102169	6	< 1
ANKRD10	Ankyrin repeat domain 10	0	47.4102169	6	< 1
CASP10	Homo sapiens caspase 10, apoptosis-related cysteine peptidase (CASP10), transcript variant A, mRNA	0	47.4102169	6	< 1
EEF1A1	Homo sapiens eukaryotic translation elongation factor 1 alpha 1 (EEF1A1), mRNA	0	47.4102169	6	< 1
ENDOGL1	Endonuclease G-like 1	0	47.4102169	6	< 1
GIPR	Gastric inhibitory polypeptide receptor	0	47.4102169	6	< 1
GUF1	GUF1 GTPase homolog (S. cerevisiae)	0	47.4102169	6	< 1
IGSF8	Immunoglobulin superfamily, member 8	0	47.4102169	6	< 1
KIFC3	Kinesin family member C3	0	47.4102169	6	< 1
LOC391636	Homo sapiens chromosome 9 open reading frame 78 (C9orf78)	0	47.4102169	6	< 1
MRPS22	Mitochondrial ribosomal protein S22	0	47.4102169	6	< 1
MTMR2	Myotubularin related protein 2	0	47.4102169	6	< 1
PCBP1	Homo sapiens poly(rC) binding protein 1 (PCBP1), mRNA	0	47.4102169	6	< 1
PRKAG2	Protein kinase, AMP-activated, gamma 2 non-catalytic subunit	0	47.4102169	6	< 1
RPL10	Homo sapiens ribosomal protein L10 (RPL10), transcript variant 1, mRNA	0	47.4102169	6	< 1
RTN4	Homo sapiens reticulon 4 (RTN4), transcript variant 1, mRNA	0	47.4102169	6	< 1
SEC31B	SEC31 homolog B (S. cerevisiae)	0	47.4102169	6	< 1
SEPT5	Septin 5	0	47.4102169	6	< 1

(*Continued*)

Genes enriched in MCF7 (breast cancer) library selection

Gene Symbol	Annotation	Enrichment with prostate specific shRNA library selection	Enrichment with MCF7 shRNA library selection	# counts in selected subset	# counts in infected subset
STK16	Serine/threonine kinase 16	0	47.4102169	6	< 1
TMEM87A	Transmembrane protein 87A	0	47.4102169	6	< 1
UBE2D3	Homo sapiens ubiquitin-conjugating enzyme E2D 3 (UBC4/5 homolog, yeast) (UBE2D3), transcript variant 1, mRNA	0	47.4102169	6	< 1
UHMK1	U2AF homology motif (UHM) kinase 1	0	47.4102169	6	< 1
ZNF579	Zinc finger protein 579	0	47.4102169	6	< 1
ADARB1	Homo sapiens adenosine deaminase, RNA-specific, B1 (RED1 homolog rat) (ADARB1) (homology with multiple targets)	0	39.50851408	5	< 1
ATXN7L2	Ataxin 7-like 2	0	39.50851408	5	< 1
B4GALT1	Homo sapiens UDP-Gal:betaGlcNAc beta 1,4- galactosyltransferase, polypeptide 1 (B4GALT1), mRNA	1.376294666	39.50851408	5	< 1
CAMTA2	Homo sapiens calmodulin binding transcription activator 2 (CAMTA2)	0	39.50851408	5	< 1
CARD8	Caspase recruitment domain family, member 8	0	39.50851408	5	< 1
CCDC75	Coiled-coil domain containing 75	0	39.50851408	5	< 1
CUL1	Homo sapiens cullin 1 (CUL1), mRNA	0	39.50851408	5	< 1
DISP2	Dispatched homolog 2 (Drosophila)	0	39.50851408	5	< 1
DYDC2	DPY30 domain containing 2	0	39.50851408	5	< 1
FLJ43963	Similar to hypothetical protein	0	39.50851408	5	< 1
FTH1	Ferritin, heavy polypeptide 1	0	39.50851408	5	< 1
KCTD3	Potassium channel tetramerisation domain containing 3	0	39.50851408	5	< 1
KIF22	Kinesin family member 22	0	39.50851408	5	< 1

(*Continued*)

Genes enriched in MCF7 (breast cancer) library selection

Gene Symbol	Annotation	Enrichment with prostate specific shRNA library selection	Enrichment with MCF7 shRNA library selection	# counts in selected subset	# counts in infected subset
KLHDC3	Kelch domain containing 3	0	39.50851408	5	< 1
LATS2	LATS, large tumor suppressor, homolog 2 (Drosophila)	0	39.50851408	5	< 1
LGALS8	Homo sapiens lectin, galactoside-binding, soluble, 8 (LGALS8), transcript variant 1, mRNA	0	39.50851408	5	< 1
LHFPL2	Lipoma HMGIC fusion partner-like 2	0	39.50851408	5	< 1
MAP4K5	Mitogen-activated protein kinase kinase kinase kinase 5	0	39.50851408	5	< 1
MGC21675	Hypothetical protein MGC21675	0	39.50851408	5	< 1
MX1	Myxovirus (influenza virus) resistance 1, interferon-inducible protein p78 (mouse)	0	39.50851408	5	< 1
NCOA4	Homo sapiens nuclear receptor coactivator 4 (NCOA4)	0	39.50851408	5	< 1
OVOS2	Ovostatin 2	0	39.50851408	5	< 1
POLL	Homo sapiens polymerase (DNA directed), lambda (POLL)	0	39.50851408	5	< 1
PUF60	Homo sapiens poly-U binding splicing factor 60KDa (PUF60)	0	39.50851408	5	< 1
RPS27L	Ribosomal protein S27-like	1.789183066	39.50851408	5	< 1
RRAD	Ras-related associated with diabetes	0	39.50851408	5	< 1
RWDD1	RWD domain containing 1	2.949202856	39.50851408	5	< 1
SETD3	Homo sapiens SET domain containing 3 (SETD3)	0	39.50851408	5	< 1
SYNJ2BP	Homo sapiens synaptojanin 2 binding protein (SYNJ2BP), mRNA	0	39.50851408	5	< 1
TADA2L	Transcriptional adaptor 2 (ADA2 homolog, yeast)-like	0	39.50851408	5	< 1

(*Continued*)

Genes enriched in MCF7 (breast cancer) library selection

Gene Symbol	Annotation	Enrichment with prostate specific shRNA library selection	Enrichment with MCF7 shRNA library selection	# counts in selected subset	# counts in infected subset
TMED5	Transmembrane emp24 protein transport domain containing 5	0	39.50851408	5	< 1
TMEM131	Homo sapiens transmembrane protein 131 (TMEM131)	0	39.50851408	5	< 1
TMEM157	Transmembrane protein 157	0	39.50851408	5	< 1
TMEM159	Transmembrane protein 159	0	39.50851408	5	< 1
UBE2Q1	Homo sapiens ubiquitin-conjugating enzyme E2Q family member 1 (UBE2Q1), mRNA	0	39.50851408	5	< 1
USP7	Ubiquitin specific peptidase 7 (herpes virus-associated)	0	39.50851408	5	< 1
VEPH1	Ventricular zone expressed PH domain homolog 1 (zebrafish)	0	39.50851408	5	< 1

Supplementary Table S4: Genes selected for siRNA based validation II

Genes enriched in prostate-specific library selection

Gene Symbol	Annotation	Enrichment with prostate specific shRNA library selection	Enrichment with MCF7 shRNA library selection	# counts in selected subset	# counts in infected subset
BIRC4	Homo sapiens X-linked inhibitor of apoptosis (XIAP), mRNA (Qiagen old name BIRC4)	129.4665976	0	10	< 1
C1orf151	Homo sapiens chromosome 1 open reading frame 151 (C1orf151), mRNA	103.5732781	0	8	< 1
C20orf7	Chromosome 20 open reading frame 7	90.62661833	0	7	< 1
ZBTB39	Zinc finger and BTB domain containing 39	90.62661833	0	7	< 1
MGC4655	B3GNT9 UDP-GlcNAc:betaGal beta-1,3-N-acetylglucosaminyltransferase 9	90.62661833	0	7	< 1
SRF	Serum response factor (c-fos serum response element-binding transcription factor)	90.62661833	0	7	< 1
RPS6KA3	Ribosomal protein S6 kinase, 90kDa, polypeptide 3	90.62661833	0	7	< 1
PPT1	Palmitoyl-protein thioesterase 1 (ceroid-lipofuscinosis, neuronal 1, infantile)	90.62661833	0	7	< 1
HNRPDL	Heterogeneous nuclear ribonucleoprotein D-like	77.67995857	0.61633282	6	< 1
CSRP2	Cysteine and glycine-rich protein 2	77.67995857	0	6	< 1
USP1	Ubiquitin specific peptidase 1	77.67995857	0	6	< 1
ZCCHC17	Homo sapiens zinc finger, CCHC domain containing 17 (ZCCHC17)	77.67995857	0	6	< 1
MRPS17	Homo sapiens mitochondrial ribosomal protein S17 (MRPS17), nuclear gene encoding mitochondrial protein, mRNA	64.73329881	0	5	< 1
HSBP1	Homo sapiens heat shock factor binding protein 1 (HSBP1), mRNA	64.73329881	0	5	< 1
RPL5	Ribosomal protein L5	64.73329881	0	5	< 1
RPL34	Homo sapiens ribosomal protein L34 (RPL34)	64.73329881	0	5	< 1
LRPPRC	Homo sapiens leucine-rich PPR-motif containing (LRPPRC), mRNA	64.73329881	0	5	< 1

(*Continued*)

Genes enriched in prostate-specific library selection

Gene Symbol	Annotation	Enrichment with prostate specific shRNA library selection	Enrichment with MCF7 shRNA library selection	# counts in selected subset	# counts in infected subset
DLEU1	Deleted in lymphocytic leukemia, 1	64.73329881	0	5	< 1
SAR1B	SAR1 gene homolog B (S. cerevisiae)	64.73329881	0	5	< 1
DNAJC14	DnaJ (Hsp40) homolog, subfamily C, member 14	64.73329881	1.232665639	5	< 1
UQCRB	omo sapiens ubiquinol-cytochrome c reductase binding protein (UQCRB), mRNA	64.73329881	0	5	< 1
UPF3A	Homo sapiens UPF3 regulator of nonsense transcripts homolog A (yeast) (UPF3A), transcript variant 1, mRNA	64.73329881	0	5	< 1
RTN1	Reticulon 1	64.73329881	0	5	< 1
SMARCE1	Homo sapiens SWI/SNF related, matrix associated, actin dependent regulator of chromatin, subfamily e, member 1 (SMARCE1), mRNA	64.73329881	0	5	< 1
PSCD3	Homo sapiens pleckstrin homology, Sec7 and coiled-coil domains 3 (PSCD3), mRNA	64.73329881	0	5	< 1
CCDC72	Homo sapiens coiled-coil domain containing 72 (CCDC72), mRNA (Homo sapiens hypothetical LOC729973 (LOC729973), mRNALength=568	64.73329881	0	5	< 1
CDC2	Cell division cycle 2, G1 to S and G2 to M	64.73329881	0	5	< 1
DALRD3	Homo sapiens DALR anticodon binding domain containing 3 (DALRD3)	64.73329881	0.61633282	5	< 1
SIGLEC5	Sialic acid binding Ig-like lectin 5	64.73329881	0.61633282	5	< 1
ZMYM5	Zinc finger, MYM-type 5	64.73329881	0	5	< 1
RPS20	Homo sapiens ribosomal protein S20 (RPS20), mRNA	64.73329881	0	5	< 1
RDH11	Homo sapiens retinol dehydrogenase 11 (all-trans/9-cis/11-cis) (RDH11), mRNA	51.78663905	0	4	< 1
S100A11	Homo sapiens S100 calcium binding protein A11 (S100A11), mRNA	51.78663905	0	4	< 1

(*Continued*)

Genes enriched in prostate-specific library selection

Gene Symbol	Annotation	Enrichment with prostate specific shRNA library selection	Enrichment with MCF7 shRNA library selection	# counts in selected subset	# counts in infected subset
C9orf119	Chromosome 9 open reading frame 119	51.78663905	0	4	< 1
PFN2	Homo sapiens profilin 2 (PFN2), transcript variant 1	51.78663905	0	4	< 1
IDI1	Homo sapiens isopentenyl-diphosphate delta isomerase 1 (IDI1)	51.78663905	0	4	< 1
SIM2	Homo sapiens single-minded homolog 2 (Drosophila) (SIM2)	51.78663905	0	4	< 1
PFTK1	PFTAIRE protein kinase 1	51.78663905	0	4	< 1
BTF3	Homo sapiens basic transcription factor 3 (BTF3)	51.78663905	0	4	< 1
SERPINB4	Serpin peptidase inhibitor, clade B (ovalbumin), member 4	51.78663905	0	4	< 1
SLPI	Secretory leukocyte peptidase inhibitor	51.78663905	0	4	< 1
NCAPG	Non-SMC condensin I complex, subunit G	51.78663905	1.848998459	4	< 1
PPM1A	Protein phosphatase 1A (formerly 2C), magnesium-dependent, alpha isoform	51.78663905	0.61633282	4	< 1
C22orf30	Chromosome 22 open reading frame 30	51.78663905	0	4	< 1
PRMT5	Protein arginine methyltransferase 5	51.78663905	2.157164869	4	< 1
LOC285741	Similar to Translationally-controlled tumor protein (TCTP) (p23) (Histamine-releasing factor) (HRF) (Fortilin)	51.78663905	0	4	< 1
C14orf147	omo sapiens chromosome 14 open reading frame 147 (C14orf147), mRNA	51.78663905	0	4	< 1
RPL36AL	Homo sapiens ribosomal protein L36a-like (RPL36AL), mRNA	51.78663905	0	4	< 1
CCDC72	Homo sapiens coiled-coil domain containing 72 (CCDC72), mRNA	51.78663905	0	4	< 1
CPEB3	Cytoplasmic polyadenylation element binding protein 3	51.78663905	0	4	< 1
SENP6	SUMO1/sentrin specific peptidase 6	51.78663905	0	4	< 1

(*Continued*)

Genes enriched in prostate-specific library selection

Gene Symbol	Annotation	Enrichment with prostate specific shRNA library selection	Enrichment with MCF7 shRNA library selection	# counts in selected subset	# counts in infected subset
GOLGA2	Golgi autoantigen, golgin subfamily a, 2	51.78663905	0	4	< 1
SULF2	Sulfatase 2	51.78663905	2.113141096	4	< 1
CCDC18	Coiled-coil domain containing 18	51.78663905	0	4	< 1
SLC39A7	Solute carrier family 39 (zinc transporter), member 7	51.78663905	2.670775552	4	< 1
ZNF146	Zinc finger protein 146	51.78663905	0	4	< 1
PNRC1	Proline-rich nuclear receptor coactivator 1	51.78663905	0	4	< 1
SMC3	Structural maintenance of chromosomes 3	51.78663905	0	4	< 1
ITPR3	Inositol 1,4,5-triphosphate receptor, type 3	51.78663905	1.284026708	4	< 1
EPC2	Enhancer of polycomb homolog 2 (Drosophila)	51.78663905	0	4	< 1
KIAA0776	KIAA0776	51.78663905	0	4	< 1
CBX7	Chromobox homolog 7	51.78663905	0	4	< 1
GPR126	G protein-coupled receptor 126	51.78663905	0	4	< 1
TRIM25	Homo sapiens tripartite motif-containing 25 (TRIM25), mRNA	51.78663905	0	3	< 1
ROD1	ROD1 regulator of differentiation 1 (S. pombe)	11.01035733	0	1	< 1
PEBP1	Phosphatidylethanolamine binding protein 1	9.634062662	0	8	1
FUCA1	Homo sapiens fucosidase, alpha-L- 1, tissue (FUCA1), mRNA	9.634062662	0	7	1
AIG1	Homo sapiens androgen-induced 1 (AIG1), mRNA	9.634062662	0	7	1
ABCC3	ATP-binding cassette, sub-family C (CFTR/MRP), member 3	9.634062662	0.528285274	7	1
MORN2	MORN repeat containing 2	8.257767996	1.643554186	7	1
HIST1H4E	Histone cluster 1, H4e	8.257767996	0	7	1
KIAA0562	KIAA0562	8.257767996	0	6	1
POLR2H	Polymerase (RNA) II (DNA directed) polypeptide H	8.257767996	0.958739942	6	1
ATP2B1	ATPase, Ca++ transporting, plasma membrane 1	8.257767996	0	6	1
RAN	GTP-binding nuclear protein Ran	8.257767996	0.880475457	6	1

(*Continued*)

Genes enriched in prostate-specific library selection

Gene Symbol	Annotation	Enrichment with prostate specific shRNA library selection	Enrichment with MCF7 shRNA library selection	# counts in selected subset	# counts in infected subset
KPNA1	Karyopherin alpha 1 (importin alpha 5)	8.257767996	2.465331279	6	1
CENPI	Centromere protein I	8.257767996	0	6	1
MAN2A2	Mannosidase, alpha, class 2A, member 2	8.257767996	0.792427911	6	1
WDR75	WD repeat domain 75	8.257767996	1.438109913	6	1
SET	Homo sapiens SET translocation (myeloid leukemia-associated) (SET), mRNA	7.569620663	0	6	1
HNRPD	Homo sapiens heterogeneous nuclear ribonucleoprotein D (AU-rich element RNA binding protein 1, 37kDa) (HNRPD),	6.88147333	0	6	1
LOC257039	Similar to 40S ribosomal protein S17	6.88147333	0	11	2
ZNF611	Zinc finger protein 611	6.88147333	0	5	1
NUDT2	Homo sapiens nudix (nucleoside diphosphate linked moiety X)-type motif 2 (NUDT2)	6.88147333	0	10	2
PLCG1	Homo sapiens phospholipase C, gamma 1 (PLCG1)	6.88147333	0	5	1
SLC25A3	Solute carrier family 25 (mitochondrial carrier; phosphate carrier), member 3	6.88147333	0	5	1
PHF20	PHD finger protein 20	6.88147333	0.739599384	5	1
STRN3	Striatin, calmodulin binding protein 3	6.88147333	0	5	1
VPS35	Homo sapiens vacuolar protein sorting 35 homolog (S. cerevisiae) (VPS35), mRNA	6.88147333	0	5	1
ATP9A	Homo sapiens ATPase, class II, type 9A (ATP9A), mRNA	6.88147333	2.711864407	5	1
SUPT16H	Suppressor of Ty 16 homolog (S. cerevisiae)	6.88147333	0.352190183	5	1
LAMC1	Laminin, gamma 1 (formerly LAMB2)	6.88147333	0	5	1
REST	RE1-silencing transcription factor	6.88147333	0	5	1
LRPPRC	Leucine-rich PPR-motif containing	6.88147333	0.821777093	5	1

(*Continued*)

Genes enriched in prostate-specific library selection

Gene Symbol	Annotation	Enrichment with prostate specific shRNA library selection	Enrichment with MCF7 shRNA library selection	# counts in selected subset	# counts in infected subset
RABL5	RAB, member RAS oncogene family-like 5	5.963943553	0	13	3
CCDC72	Coiled-coil domain containing 72	5.780437597	0.92449923	4	1
RPL22L1	Ribosomal protein L22-like 1	5.505178664	0	8	2
HAT1	Histone acetyltransferase 1	5.505178664	0	16	4
SEC61G	Sec61 gamma subunit	5.505178664	0	8	2
IPO9	Importin 9	5.505178664	2.275690411	8	2
DHX35	DEAH (Asp-Glu-Ala-His) box polypeptide 35	5.505178664	0	10	3
POLR2K	Homo sapiens polymerase (RNA) II (DNA directed) polypeptide K, 7.0kDa (POLR2K), mRNA	38.83998	0	3	< 1

Supplementary Table S5: New regulators of androgen-responsive genes found in the functional screening

Gene ID	Gene Name
CD9	CD9 molecule
DYDC2	DPY30 domain containing 2
RABL5	RAB, member RAS oncogene family-like 5
SMARCE1	SWI/SNF related, matrix associated, actin dependent regulator of chromatin, subfamily e, member 1
AIG1	androgen-induced 1
CAPZB	capping protein (actin filament) muscle Z-line, beta
C14orf147/ SPTSSA	chromosome 14 open reading frame 147/ serine palmitoyltransferase, small subunit A
HAT1	histone acetyltransferase 1
IGSF8	immunoglobulin superfamily, member 8
MRPS17	mitochondrial ribosomal protein S17
NUDT2	nudix (nucleoside diphosphate linked moiety X)-type motif 2
PUF60	poly-U binding splicing factor 60KDa
POLR2H	polymerase (RNA) II (DNA directed) polypeptide H
RTN1	reticulon 1
SUPT16H	suppressor of Ty 16 homolog (S. cerevisiae);
ZNF146	zinc finger protein 146
PCS5/PCSK5	protein convertase subtilisin/kexin type 5
RPL5	ribosomal protein L5
ASCC3L1/ SNRNP200	small nuclear ribonucleoprotein 200kDa (U5)
RPL36AL	ribosomal protein L36a-like

Supplementary Table S6: Genes, differentially regulated by IGSF8 knockdown and activation of androgen receptor (See supplemental Excel table). Results represented in 4 worksheets. (1) IG +>1.4 R18 > 1.4 - genes up regulated 1.4 fold or more by siRNA IGSF8 and R1881 treatment. (2) IG - < 1.5 R18 - < 1.5 - genes down regulated 1.5 fold or more by siRNA IGSF8 and R1881 treatment. (3) IG - < 1.5 R18 + > 1.5 - genes down regulated 1.5 fold or more by siRNA IGSF8 and up regulated 1.5 or more by R1881 treatment. (4) IG + > 1.5 R18 - < 1.5 - genes up regulated 1.5 fold or more by siRNA IGSF8 and up down 1.5 or more by R1881 treatment.

Supplementary Table S7: Target sequences of siRNAs used for the knockdown of the indicated genes

Gene	*siRNA*	*Target sequence*
AIG1	Hs_AIG1_2	CAGATGTTTCTCATTGCATAA
ASCC3L1	Hs_ASCC3L1_1	ACCCAGGTGTTTAACACTGTA
C14orf147	Hs_C14orf147_1	TACATTAAATTCAAAGAGAAA
CAPZB	Hs_CAPZB_4	AAAGCTGGAGGTGGAAGCCAA
CD9	Hs_CD9_4	CCGCGAGATGGTCTAGAGTCA
DYDC2	Hs_MGC16186_4	CCGAGTCAAAGGAAAGAGAAA
HAT1	Hs_HAT1_3	AAGACTAATTAGCCCATATAA
IGSF8	(1) Hs_IGSF8_2	TGGGCCCAGATTGCAGAGAAA
IGSF8	(2) Hs_IGSF8_7	CCGGTCAGACTTGGCCGTGGA
MRPS17	Hs_MRPS17_8	CAGGAAAGCCCTGTGCTGGAA
NUDT2	Hs_NUDT2_1	TACCTTGTACTTTATAAATAA
PCSK5	Hs_PCSK5_1	CCGCGAGATGGTCTAGAGTCA
POLR2H	Hs_POLR2H_4	ACATTCAAATTTACCCTGTA
PUF60	(1) Hs_SIAHBP1_4	CTCCAGGTCAATGGCCAGCAA
PUF60	(2) Hs_SIAHBP1_1	CTCCAGGTCAATGGCCAGCAA
PUF60	(3) Hs_SIAHBP1_2	CTGGGACTCCGTCACCATGAA
RABL5	Hs_RABL5_7	CCGGATGGAATTCATAAAGTA
RPL36AL	Hs_RPL36AL_3	AAGGAGGCGCTATGATCGGAA
RPL5	Hs_RPL5_3	ATGGAGGAGATGTATAAGAAA
RTN1	Hs_RTN1_2	AACCACTAAAGTATAGAGAAA
SMARCE1	Hs_SMARCE1_9	AAGATAGTTTGTGACACATAA
SUPT16H	Hs_SUPT16H_5	CAGCATATGCATGACCGAGAT
ZNF146	Hs_ZNF146_3	CAGAGAAGCCTTATAAATGTA

Supplementary Table S8: Primer sequences for QPCR of the indicated genes

Gene	*Sense*	*Antisense*
AIG1	TTATTAAGGTGCTGCGGATGG	GGTCTGGTGTGAGGGCATTT
AR	GCAGGAAGCAGTATCCGAAG	CGTTGTCAGAAATGGTCGAA
ASCC3L1	CATCCAACGGCAGAGGTTCT	AGCGTGCTGTCACTTAGCTT
C14orf147	AGCGGACGGTGTTCAATTCC	CCTCTGATCCTGGTCGCATC
CAPZB	GTCACCATGGAGTAACAAGT	CCAGAGGTAGACAGATGAGA
CD9	AGGTCTTCGACAATAAATTC	AGGGATGTAAGCTGACTCTIGSF
DYDC2	ATTCTTATCACCTTGCCTAC	CAGGTAGTTAGTTTCCATCC
FKBP5	TGAGGAAACGCCGATGATTG	CCTTGATGACTTGGCCTTTG
GAPDH	AGGTGAAGGTCGGAGTCA	GGTCATTGATGGCAACAA
GL3 luciferase	AAGCGAAGGTTGTGGATCTG	TGTCAATCAAGGCGTTGGTC
HAT1	CGGGTTGATTCGTCCTTCCT	CACCAAATCCCGCCATTTCC
IGSF8	TGCTGCTGCTAATGCTAGG	CACATTGCAGGAGATGGAGAC
KLK2	GCTGCCCATTGCCTAAAGAAG	TGGGAAGCTGTGGCTGACA
KLK3	AGCTGTGGCTGACCTGAAAT	GTCCTCACAGCTGCCCAC
MRPS17	TCTTGGCGGAGGTGACCAAA	AATCCATCTGGCATGGACGG
NUDT2	TTTCTGCCTCAACCACGGAC	CAAACGCACCCATTCCAGAAG
PCSK5	ACTATGGCACAGAGGATTAT	ACACAGATCCTGGTATTGTT
POLR2H	AACACGCCTGCTGAGATTGA	ACGTACGCAGAGCTGAAACA
PUF60	ATGAGCATCTCGGGCAGTAG	ACTTGCCACACTCCTCTGTC
RAB5L	AGTTGAGCCTCTGAAGCTGT	GGACCTCCAACTCAGTTCCAT
RPL13A	AGATGGCGGAGGTGCAG	GGCCCAGCAGTACCTGTTTA
RPL36AL	GGCGCTGTTTCTCGTTTTCA	GATCATAGCGCCTCCTTCCC
RPL5	CAGCGTATGCACACGAACTG	ACCTATTGAGAAGCCTGCGG
RTN1	AGAACGAAGCGGTGACGC	TGCCACACCTGTGGATGC
SHH	CCAAAGCGTTCAACTTGTCC	TTTAAGGAACTCACCCCCAA
SMARCE1	GCAGAAAGGTCTGGGACCAA	CAAGGTACGCGGGGGAATTA
SUPT16H	CGGAATGGCCACCTTTTGTG	ACCCGCTCAAAGTGGATCAG
TMPRSS2	TACCACAGTGATGCCTGTTC	CGGCTTGAGTTCAAGTTGAC
ZNF146	AGAAGGTAATTCAGTACCAGCCT	CATATTAACTCCTACTTTCGCGCC
UGT2B7	TCCCATCAAATCTCCACAGA	GGTGTTTTCTCTGGGGTCAA
UGT2B15	TGATCTGAAGAAGTGGGACCAG	CCATTTCCTTAGGCAGGGGT
UGT2B17	CAAGCATATGATCTGAAGAAGTGGG	AGTGGGTCTTCCTAGAACTTCAC
